# Trends and Commonalities of Approved and Late Clinical-Phase RNA Therapeutics

**DOI:** 10.3390/pharmaceutics17070903

**Published:** 2025-07-12

**Authors:** Maxime Tufeu, Christophe Herkenne, Yogeshvar N. Kalia

**Affiliations:** 1 School of Pharmaceutical Sciences, University of Geneva, 1211 Geneva, Switzerland; 2 Institute of Pharmaceutical Sciences Western Switzerland, University of Geneva, 1211 Geneva, Switzerland; 3 Debiopharm Research and Manufacturing, 1920 Martigny, Switzerland

**Keywords:** nucleic acids, delivery, targeting, topical, local administration, endosomal escape

## Abstract

**Background/Objectives:** After many years of research and the successful development of therapeutic products by a few industrial actors, the COVID-19 vaccines brought messenger RNAs, as well as other nucleic acid modalities, such as antisense oligonucleotides, small interfering RNA, and aptamers, into the spotlight, eliciting renewed interest from both academia and industry. However, owing to their structure, relative “fragility”, and the (usually) intracellular site of action, the delivery of these therapeutics has frequently proven to be a key limitation, especially when considering endosomal escape, which still needs to be overcome. **Methods**: By compiling delivery-related data on approved and late clinical-phase ribonucleic acid therapeutics, this review aims to assess the delivery strategies that have proven to be successful or are emerging, as well as areas where more research is needed. **Results**: In very specific cases, some strategies appeared to be quite effective, such as the N-acetylgalactosamine moiety in the case of liver delivery. Surprisingly, it also appears that for some modalities, efforts in molecular design have led to more “drug-like” properties, enablingthe administration of naked nucleic acids, without any form of encapsulation. This appears to be especially true when local administration, i.e., by injection, is possible, as this provides de facto targeting and a high local concentration, which can compensate for the small proportion of nucleic acids that reach the cytoplasm. **Conclusions**: Nucleic acid-based therapeutics have come a long way in terms of their physicochemical properties. However, due to their inherent limitations, targeting appears to be crucial for their efficacy, even more so than for traditional pharmaceutical modalities.

## 1. Introduction

**Figure 1:**
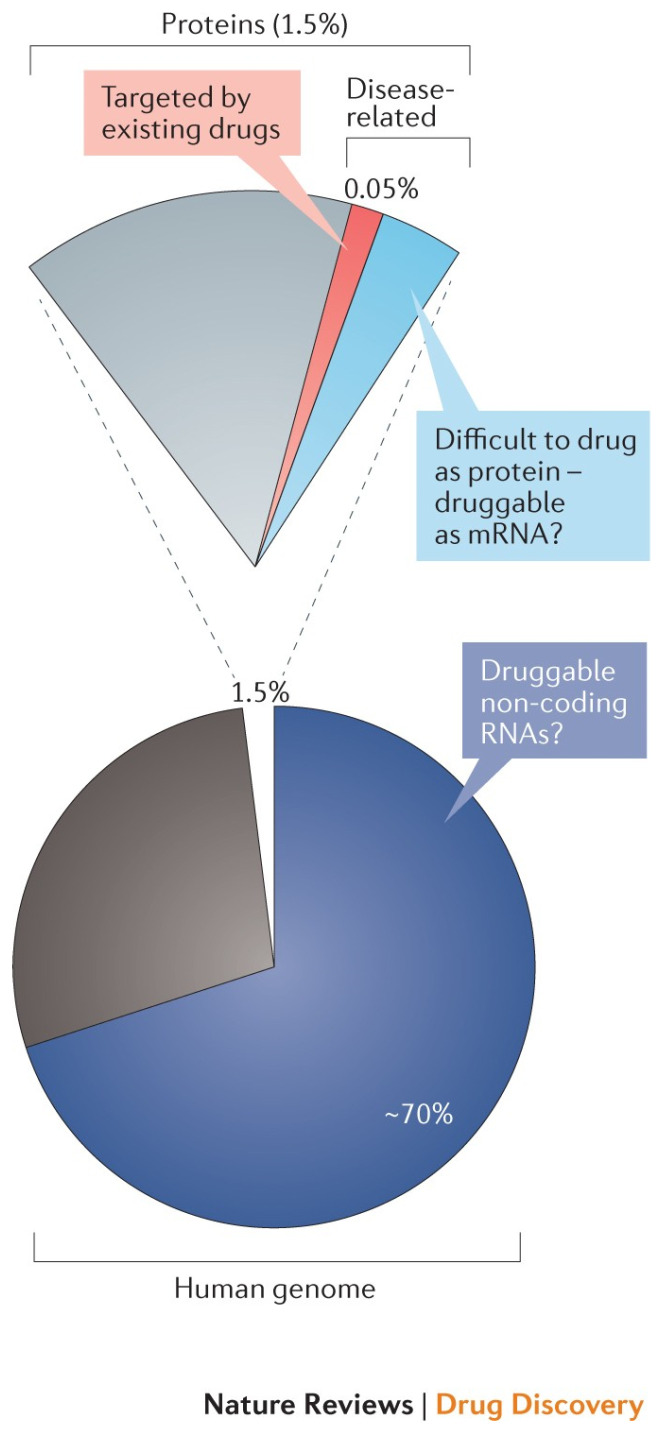
Drugging the RNA genome. Reproduced with permission from Warner et al., 2018 [5]. This figure illustrates the total human genome as composed of non-coding regions in grey, regions coding for non-coding RNAs in dark blue (estimated at 70% of the genome), and of genes coding for RNAs, which in turn code for proteins (estimated to be 1.5% of the genome). Of these protein-coding genes, the authors estimate that 15% are associated with diseases. In addition, it is estimated that the proteins that are currently “drugged” by an approved product correspond to 0.05% of the total genome. Hence, considering targets in the RNA genome offers a wider array of therapeutic options than the more traditionally explored proteome.

After years of research, development, and even commercialization of various nucleic acid-based products, the rapid and effective response to the COVID-19 pandemic put ribonucleic acid (RNA)-based therapeutics in the spotlight and highlighted their potential as therapeutic agents. Although the development of mRNA vaccines was a great achievement, as these nucleic acids enable the exposure of the immune system to proteins produced by cells in situ and are of particular interest for proteins that would otherwise be more difficult to produce and/or to administer, they only represent the tip of the iceberg regarding the many possibilities that RNA-based therapy has to offer. Indeed, if the potential of RNA therapeutics is deemed “limitless” by some [1,2,3], this is because of the existence of many different RNA modalities. As each of these modalities has different functions in the cell, targeting RNA or using RNA-therapeutics is believed to enable the “drugging” of the (until now) “undruggable” [4,5]. This is because the usual strategy is to target a protein, e.g., an enzyme or a membrane receptor, whose conformation has to contain some kind of “pocket” (or “lock”) for the drug (or “key”) to interact with it [6]. However, in more recent years with the advance of biology and cellular biology, we not only know that traditionally “druggable” and “undruggable” proteins alike are translated from mRNA, but also that not all RNAs are coding. In this context, the proteome could be considered the product of a small fraction of the bigger RNA genome (Figure 1), which broadens the scope of therapeutic targets.

**Figure 2:**
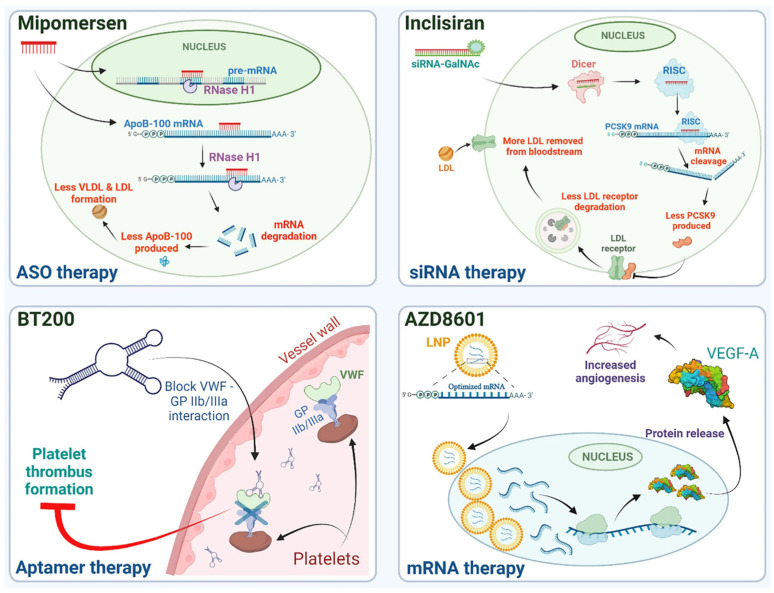
Examples of different RNA modalities. Reproduced with permission from Bejar et al., 2022 [7]. The most common modalities of therapeutic nucleic acids allow different forms of control over proteins that may be disease-related, more so than using traditional therapies that act at the protein level. For example, aptamers (**bottom left**) can bind to receptors in a “classical manner” to act as antagonists, while siRNAs (**top right**) and ASOs (**top left**) can lead to mRNA degradation and thus inhibit activity of the protein that would have been translated. ASOs can even act in the nucleus to provide alternative splicing of mRNAs that lead to modified proteins. Finally, mRNAs can increase the amounts of a given protein that are produced, leading to increased activity.

Indeed, Figure 1 shows that ~1.5% of RNA codes proteins, of which perhaps 15% are believed to be disease associated. More importantly perhaps, one can also see that it is thought that 70% of all RNAs are non-coding but could be druggable, which represents a major fraction of the RNA genome and thus many different cellular functions. Therefore, by using RNA-based products or drugging the RNA genome, in theory, a larger number of diseases could be treated, using many different biological mechanisms. Indeed, as stated before, many different modalities of RNA have been identified, all of which have a different role in cellular processes and could, therefore, be targeted or used in different ways as therapeutics agents. Examples of the most common of these modalities currently under development or in commercial products are presented in Figure 2.

The top left panel presents the mechanism of action of Mipomersen, an antisense oligonucleotide (ASO). These nucleic acid sequences are usually short, single strands between 10 and 30 bases in length [8]. Although they are originally based on a DNA structure, these oligonucleotides can be composed of a mixture of ribonucleotide, deoxyribonucleotide, or completely synthetic analogues. ASO can have an effect on different RNA modalities in the nucleus or in the cytoplasm [9], and in different ways, such as alternative splicing, the modulation of post-transcriptional modifications, degradation through RNase H, or even the inhibition of their target activity through pairing to their target [10,11].

The top right panel shows the mechanism of action of Inclisiran, an FDA-approved siRNA therapeutic; siRNAs are short, double-stranded (guide and passenger strands) oligonucleotides, with a length of around 21 bases [12,13]. Unlike ASO, they cannot exert any effect in the nucleus. Once inside the cell, the two strands separate, and the guide strand enters the RNA-induced silencing complex (RISC) protein complex. The siRNA-loaded RISC will then pair with the target mRNA by base complementarity and lead to its cleavage. Closely related to siRNAs are micro RNAs (miRNAs) [12], which mostly differ by their endogenous synthesis and by the fact that their pairing is not 100% complementary to their target. Moreover, miRNAs can repress their target mRNA translation without leading to its cleavage unlike siRNAs.

BT200 (Figure 2, bottom left) is an example of an aptamer. This type of oligonucleotide has impressive binding capabilities due to the diversity of sequences and conformations that they can adopt. They are able to bind multiple targets—from metal ions to supramolecular structures—with a tremendous variability in their target’s chemical nature (e.g., sugars, proteins, nucleic acids …) [14]. Aptamers can be used to inhibit or activate their target once bound or used as a targeting agents for another actives [15]. Aptamers have also been used as a targeting agent for more complex drug delivery systems (DDS) [16].

Finally, the last RNA modality presented in Figure 2 is mRNA, which is perhaps the most widely known therapeutic nucleic acid format. mRNAs are single-stranded, relatively large macromolecules, typically around 1000 bases [17]. They appear to be mostly linear but do present some domains with secondary structures due to base pairing, which can have important effects for translation [18]. Once mRNAs reach the cytoplasm, they interact with ribosomes to be translated into proteins [19]. This is especially interesting, because it allows the production of proteins in situ, taking advantage of the whole cellular machinery for post-translational modifications, which can be hard to replicate in vitro. Therefore, mRNA can be used to supplement a missing or faulty protein but also to produce antigens in the body, thus eliciting an immune response, as was the case for the COVID-19 vaccines.

These modalities represent the most described RNA technologies, but they are only the most common examples of the many possibilities that nucleic acids can offer. Indeed, many more are described in the literature, at different stages of research and development, such as self-amplifying RNA (saRNA), circular RNA (circRNA) [20], and single guide RNA (sgRNA) used for CRISPR/Cas9 gene editing [21], for example.

Besides the many therapeutic opportunities presented above, compared to other active pharmaceutical ingredients, RNAs can offer major advantages in terms of synthesis and the potential speed of development, as seen for COVID-19 vaccines [22]. Indeed, unlike proteins, RNA can be synthesized without the need for cell culture by in vitro transcription. This greatly shortens the time needed to obtain nucleic acids, both in development and in manufacturing, since no cell lines have to be generated and cloned, and no upstream steps prior to purification steps are needed.

However, despite these advantages, their use as therapeutic agents remains fairly new, with an appreciable lag time between their discovery and use in biological research (in which they allow numerous cellular functions and mechanisms to be modulated and investigated) and their use as therapeutics. This is illustrated by ASOs and siRNAs, for which 20 years separate their respective discovery and their use as approved therapeutic agents [23]. A more striking example of that delay between discovery and use in therapeutics can be seen for mRNA, with Malone’s work on mRNA transfection in eukaryotic cells, using cationic liposomes, to express protein as early as 1989 [24,25]. This paper was published more than 30 years before the first mRNA product was approved in 2021 (in 2020, mRNA vaccines received an emergency approval, whereas full approval came a year later), in which case an argument could be made that the COVID-19 crisis was an extreme accelerating factor [26]. Another interesting aspect of the rise of ribonucleic acid-based pharmaceuticals is the drought of new products that can be seen for almost a decade at the beginning of the 2000s, highlighting the challenges that the industry had to overcome to harness the potential of nucleic acids. Indeed, among the 16 products that have received FDA approval until now (including Fomivirsen whose approval was withdrawn in 2001), two products were approved in the late 1990s to early 2000s, only for the subsequent products—Mipomersen and Defibrotide—reaching the market almost ten years later. It is also important to note that ribonucleic acid-based products can present side effects after administration to patients, which can be severe [27]. These side effects can be administration route-related (such as injection site inflammation), due to the delivery systems, which will be discussed below, or a reaction to the RNAs themselves [28]. Indeed, if RNAs are part of normal metabolism, their presence in unexpected compartments can be detected by the immune system as a threat and thus lead to immune response [29].

Taken together, these elements, as well as insights from major actors in the field and industries [2,30,31], show that despite the previously mentioned advantages and strengths of ribonucleic acid-based products, the use of these promising agents as therapeutics has followed a challenging path. To name the main hurdles that have been—or still need to be—overcome, one can mention toxicity and immunogenicity of both the nucleic acids and components of the drug delivery systems, stability during manufacturing, storage, and in the organism, as well as efficient delivery in vivo, and finally, regulatory considerations.

This review focuses on delivery with particular emphasis on RNA-based products in late clinical development (phases 2/3 and 3) and products that have already been approved as of January 2023. The goal was to explore the delivery aspects of RNA therapeutics and more specifically the strategies used to address the delivery-related challenges mentioned above that enabled these products to reach later development stages.

The first part of the review presents the delivery routes and target organs of siRNA and ASOs for products already on the market, as well as for products in late clinical development. Both modalities will be explored in parallel, as the evolution of their development, such as the CMC strategies that were used, is rather similar in nature. The second part focuses on mRNA-based therapeutics, either already marketed or in late-stage clinical development. The delivery of this modality is explored separately, as its problematics and challenges are rather different to those for siRNAs and ASOs, impacting formulation, delivery routes, current therapeutic area, and seemingly, further development leads. Finally, the delivery of aptamers is explored in a separate section, as none of the aptamers commercialized or in late-stage clinical development have an intracellular target, as opposed to the other modalities.

## 2. Antisense Oligonucleotides and siRNA

**Figure 3:**
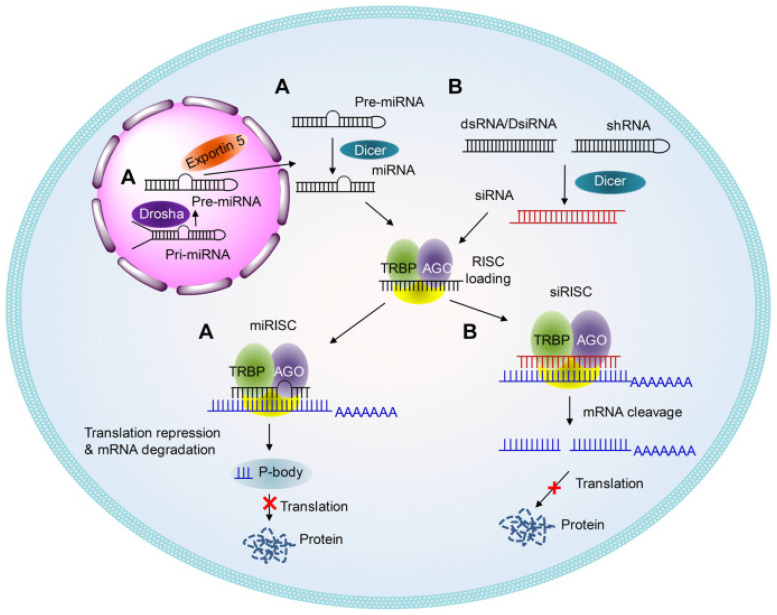
siRNA and miRNA pathways. Reproduced with permission from Hu et al., 2020 [32]. A: The figure shows the miRNA pathway, in which the complementarity of the RNA strand and its targeted mRNA (blue) is not complete. It interacts with a cytoplasmic protein complex known as RISC and the mRNA-RISC interaction can lead to the modulation of protein expression (indicated by X) and mRNA degradation. B: siRNAs (red) are duplex nucleic acids, of which one strand can interact with the RISC complex. The RISC complexed single strand can then pair with a target mRNA leading to its degradation and thus inhibiting translation (red cross). This decreases the amount of protein produced and, therefore, the activity of said protein.

Antisense oligonucleotides (ASOs) are composed of a single strand of around 20 nucleotides, while small interfering RNAs (or short interfering RNAs, silencer RNAs, siRNAs) are composed of two strands of roughly the same length, making both modalities rather “small” nucleic acids. Both can be used to modulate the expression of target genes at the mRNA level; although, the underlying mechanisms are different. Indeed, the latter requires interactions with several plasmatic proteins leading to mRNA degradation (Figure 3).

**Figure 4:**
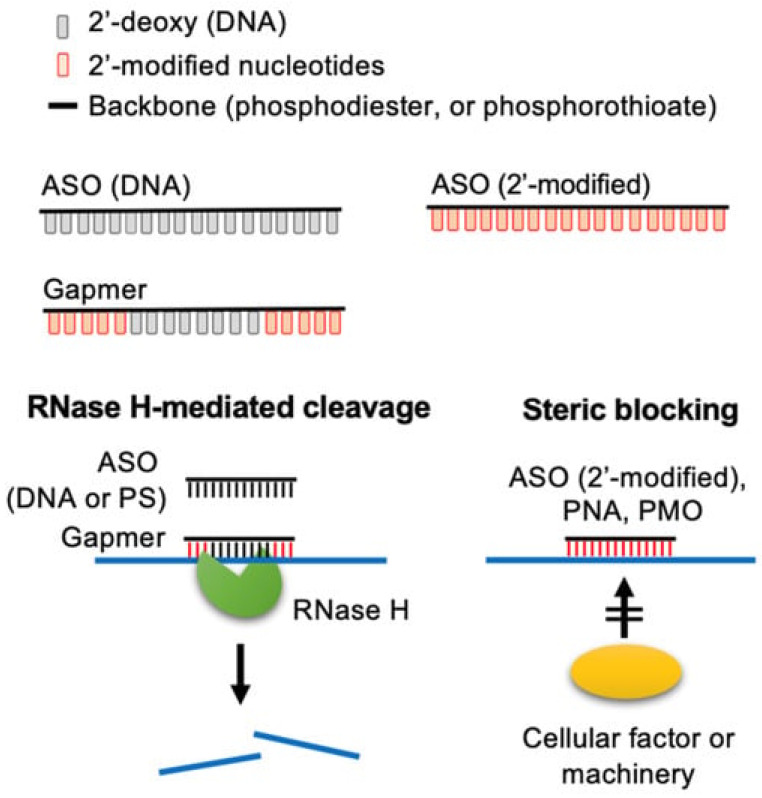
Main ASO activity pathways and example of corresponding chemical modifications. Reproduced with permission from Tarn et al., 2021 [34]. In this figure, the authors show how the structure of an ASO can lead to a different mechanism of action. Indeed, for RNase H to cleave an mRNA, it needs to recognize a DNA/RNA duplex. Therefore, by selectively modifying the 2′ position of nucleotides at the extremities of an ASO, leaving a DNA “gap” in the center, cleavage by RNase H is possible. However, if all nucleotides of an ASO have their 2′ position modified, the DNA/RNA duplex cannot be recognized by RNase H. In this case, the mRNA is not degraded, but the ASO can have an activity by preventing other cellular factors from interacting with the mRNA by steric hindrance.

In contrast, the effect of ASOs relies on the direct interaction of the oligonucleotide with its target mRNA to form a pair. This pair can then be recognized by RNase H and lead to the degradation of both the ASO and target mRNA or can sterically block interaction of the mRNA with other cellular machinery, leading to the alternative splicing or transient silencing of the mRNA (Figure 4). As implied by the alternative splicing capabilities of ASOs, this also means that unlike siRNA, they can have an activity inside the nucleus [33].

### 2.1. Tissue Targeting and Drug Delivery Systems

This section focuses on the relations between target tissues, administration routes, and drug delivery strategy. A comparison is made between products already on the market and products currently in the late clinical phase, which may have benefited from later scientific advances and an improved understanding of the requirements for nucleic acid-based therapeutics.

#### 2.1.1. Approved Products

The target organs and corresponding routes for approved ASO- and siRNA-based products are presented in Table 1 and Table 2, respectively.

As one can see, although the ASOs have been deployed to target diverse tissues, siRNAs have only targeted the liver. For ASOs, the liver is also well represented; although, the main target appears to be the muscles, followed by the central nervous system and the eye.

**Figure 5:**
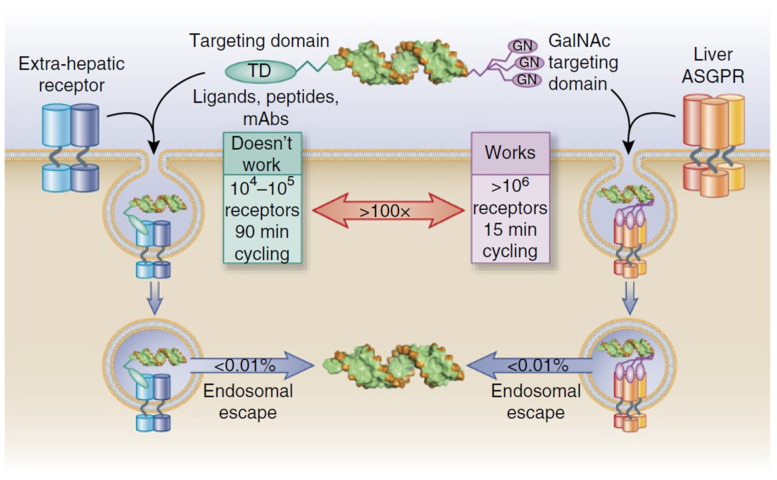
Comparison between ASGPR and other receptors mediated cellular uptake. Reproduced with permission from Dowdy, 2017 [45]. The ASGPR receptor is a very singular example of transmembrane receptor mediated uptake. Although the main barrier to cytosol activity remains the endosomal escape, binding to this receptor leads to increased cytosol concentration thanks to two main characteristics of the receptor. First, ASGPR is particularly densely expressed at the surface of hepatocytes, up to a hundred times more than other receptors, which means that more targeted siRNA can bind to the same number of hepatocytes compared to non-ASGPR expressing cells. Furthermore, the turnover of this receptor is higher than most, with a cycle estimated to be six times shorter than more common receptors. This means that each time a receptor is bound to a targeted siRNA leading to endocytosis in a hepatocyte, the delay before this receptor is available at the cell surface again to bind a new siRNA is shorter than other receptors. Combined, these two properties lead to more siRNAs reaching the endosomal escape bottleneck. Therefore, if the proportion of siRNAs that undergo endosomal escape remains the same, the number of siRNAs that go through the bottleneck is higher.

The significance of the liver is to be expected, as this organ is one of the main sites of the accumulation of naked ASOs in the body [41]. However, for siRNAs, the preponderance of liver-targeting products is mostly due to the strategies that were developed to efficiently target this organ. Indeed, if the approved ASOs rely on passive biodistribution to accumulate in the liver, siRNAs use two different DDS. To begin with, one of these products, Patisiran, is formulated in lipid nanoparticles (LNP), for which liver tropism is well described and linked to their surface composition, leading to apolipoprotein E binding and “LDL-like” metabolization [42]. Regarding the four other siRNAs, a conjugation strategy has been used, based on an N-acetylgalactosamine (GalNac) moiety. This ligand gives a tremendous advantage in cellular uptake in hepatocytes owing to the particular properties of the asialoglycoprotein receptor (ASGPR) to which it binds [43,44], including high receptor density and turnover. Nevertheless, this receptor/moiety pair is rather particular, as its really interesting properties in terms of targeting are unfortunately not usually found in other tissues (Figure 5) [45]. Readers are encouraged to explore the history [46,47] of the discovery of this mechanism for siRNA and ASO delivery, as its implication in the perceived importance of these modalities as human therapeutics was tremendous. It is also a great example of academia–industry collaboration, as the University of Leiden in collaboration with Alnylam led to major progress in using this moiety for siRNA delivery in humans [48,49].

It must be noted that if the LNPs evoked above do not profit from the high turnover of the ASGPR, their endosomal escape (which is often considered as the main hurdle in nucleic acid delivery [50,51]) is rather good thanks to the fusogenic lipids that they contain. This difference in endosomal escape capabilities could explain the ten-fold lower dose of Patisiran compared to other GalNac-targeted products, such as Givosiran; although, this could also stem from better IV bioavailability or the higher toxicity of the LNP carrier (which will be further discussed later). It is also interesting to see that for those liver-targeted products, the subcutaneous route is preferred, the only product injected IV being the only one formulated as an LNP. This could indicate that a LNP formulation compatible with SC injection might not yet have been achieved, which could be hypothesized to be linked to stability issues at higher concentrations. Overall, it is very interesting to see that whether it is by simple tropism or by targeted delivery strategies, both siRNAs and ASOs tend to be relatively easy to deliver to the liver. Indeed, this organ is central to many metabolic pathways and produces a vast variety of proteins, which are then distributed to the rest of the body. The accumulation of active pharmaceutical ingredients that are able to modulate protein synthesis in this organ can, therefore, affect many physiological functions and, hence, pathologies in distant tissues.

For the eye, CNS, and muscle-targeted ASOs, no specific DDS seems to be used. For the first two targets, injections are made locally, most likely due to these organs being quite isolated histologically and difficult to reach through the systemic circulation. Although this makes for rather invasive administration routes, this can also explain that no targeting or DDS was needed. For muscle-targeted products, an IV injection is used. If the use of a route that reaches the systemic circulation is mandatory for diffused tissues, such as muscles, it is interesting to see that no particular DDS was used to protect the ASOs from nucleases or to avoid triggering an immune response. This will be discussed later in this review, as this is an interesting example of the importance of chemical modifications for drug delivery in the nucleic acid field.

#### 2.1.2. Late Clinical-Phase Products

To better understand the trends in the delivery of these small nucleic acids, let us now explore the products that are currently in late-stage clinical development (phase II/III and phase III). As for the approved treatments described previously, the targeted organs and corresponding routes are presented for these products in Table 3 and Table 4.

It is striking to see that the number of different target organs and administration routes is much increased for the candidates in late-stage development. Indeed, the therapeutic use of siRNAs has expanded outside of the liver with indications in eye and kidney diseases. For ASOs, the spectrum of targets is even broader, with indications in GI tract diseases and tumoral lesions.

Nevertheless, for both modalities, one can see that the liver remains the most targeted organ. An interesting change, however, is the DDS used for those liver-targeted treatments. Indeed, if for siRNAs, the use of GalNac remains the norm, for ASOs, it is now used by four out of the five oligonucleotides used to silence protein expression in the liver (it must be noted that the DDS for the last of these ASO could not be found in the literature at the time of writing). These elements really underline the tremendous impact that this conjugation-based targeting had on liver delivery of small nucleic acids, some even going as far as saying that “liver hepatocyte delivery has been solved” [51].

Another key point is the progression of local administration routes. If the main administration route for approved products is via the systemic circulation, for these more recent products, the trend is reversed. It appears quite clearly that the local delivery of these small nucleic acids increased as a proportion of the number of products, as did the variety of administration routes used. As one can see, many of these local administration routes are rather invasive, involving intraocular, intrathecal, or intratumoral injections. However, in these newer products, some interesting routes in terms of patient comfort and compliance are being explored, such as oral or ocular (topical) administration. Furthermore, none of these locally injected nucleic acid products are using a targeting strategy (whether it is formulation or conjugation), which raises the question of which came first? The local injection, or the lack of viable targeting strategy? The answer might depend on the local route considered. Indeed, for these “patient-friendly” routes, using a local administration strategy deprived of targeting agents makes sense, whereas one could argue that if a targeting moiety (such as other sugars, peptide, or antibodies) was sufficient to efficiently cross the BBB and avoid intrathecal injection, for example, it would be chosen over the complicated intervention.

It is also interesting to note that one of the new target organs observed in late clinical products is the kidney, and that the corresponding siRNA does not use a particular DDS, despite its intravenous injection. Indeed, thanks to its filtering role, this organ is part of the “natural biodistribution sites” of siRNAs [89]. However, the cellular uptake capabilities of this organ are not expected to be high. This could explain the relatively high doses used in the clinical trial, which are 10 mg/kg [79], 3 to 4 times more than other naked, GalNac-conjugated siRNAs, which are administered SC.

In terms of DDS, except for previously mentioned conjugation strategies, the use of advanced formulation technologies is rather scarce, with only three reported cases. However, these three formulations technologies are quite interesting. Indeed, of all siRNAs considered in this review, only one product is formulated in LNPs rather than a simple solution—Patisiran, the first siRNAs targeted to the liver. It is interesting, as it reinforces the idea that once GalNac conjugation was brought to the market, it overshadowed any other strategy for delivery to hepatocytes.

In the ASO world, the first product of interest in terms of DDS is ION363, which uses an adeno-associated virus delivery system. These DDS are efficient for gene delivery but can have some drawbacks, such as their complexity of development and characterization as well as their immunogenicity [90]. Nevertheless, this viral product is meant for intrathecal injection, which surely profits from the brain’s immune privilege [91]. A point to consider though when using this kind of “biological” DDS (such as exosomes, viral vectors…) for RNA-based products is the fact that these systems require complex manufacturing steps and extensive characterization. This added complexity can in a way defeat one key advantage of these smaller nucleic acid therapeutics, that is, their cell-free production, which can greatly simplify production and characterization.

Finally, the last DDS used for ASOs is surprisingly a rather classical one, namely a delayed release tablet for oral administration. Although this may look trivial, this is an interesting element, as it implies that smaller nucleic acids can be used in more traditional formulations, and thus manufacturing processes, and can be exposed to the relatively harsh conditions of the intestinal lumen while a priori keeping their activity. While this appears to be tremendous progress for patient comfort, compliance, and accessibility to treatments, it is important to note that this delivery system may be possible thanks to the specificities of the disease target. Indeed, Mongersen administered per os is used to treat Crohn’s disease, by lowering the local expression of a protein. Therefore, in this case no systemic effect is needed for this ASO, so no permeation across the intestinal barrier is needed, solely penetration and accumulation. Furthermore, while Crohn’s disease can affect any part of the GI tract, most of the patients suffer from ileum and colon inflammation, which are the segment of the intestine in which the oral tablets are designed to deliver the ASO [92]. It is important to highlight that different segments of the GI tract might display different properties with regard to pH, enzymatic activity, and absorption [93]. Therefore, while this is already impressive progress in nucleic acid-based product delivery, it is complicated to really assess from this single product whether an oral delivery system for small nucleotides could allow for systemic treatments or even for local treatments in other parts of the intestine.

Overall, based on the evolution of their delivery strategies between products that are marketed and still in development, ASOs and siRNAs appear to have gained more and more drug-like properties with regard to their delivery. Indeed, many different administration routes appear to be conceivable, without the need for encapsulation to protect them from degradation by nucleases or from triggering an immune response. This is important to point out, as considering the immunogenicity and fragility of these actives, it highlights the central role of chemical modifications for the drug delivery of ASOs.

### 2.2. Structure-Based Approach to Delivery

As alluded to in the previous section, short nucleic acids, such as ASOs and siRNAs, are widely used as therapeutics as “plain” solutions, without any form of encapsulation to protect them from degradation by nucleases or to limit the immunological response that extracellular genomic material can cause. The main reason for this is the development and application of many different forms of chemical modification strategies. Mainly directed at the backbone of the nucleic acid and at the 2′ carbon of the pentose, these modifications have allowed for major improvements of the properties of both ASOs and siRNAs. Altering the repeating phosphodiester motif of the backbone increased resistance towards nucleases as well as enhancing cellular uptake in some cases, while 2′ modification also led to improved nuclease resistance, as well as higher cellular uptake, reduced toxicity (which can be brought about by some backbone modifications) and improved binding to the target [94,95,96,97]. It should be noted that these modifications can also target the bases themselves or the 3′ and 5′ positions of the strands (such as inverted nucleosides); however, their effect is limited in terms of delivery.

Although these chemical modifications share the same aim in both ASOs and siRNAs (enhanced stability, lowered immunogenicity, overall better drug-like properties), these two different modalities use different cellular pathways and molecular machinery. This means that one cannot apply the same modification strategies to both modalities to ensure activity. Indeed, as stated earlier, ASOs are small polynucleotides, which can have different effects depending on their structure [34,98]. In all cases, these ASOs interact with their target RNA through Watson and Crick base pairing to either induce RNase H-mediated hydrolysis or to prevent interactions with other molecules, such as enzymes, e.g., by steric blocking. In both cases, the chemical structures needed in the target–ASO pair for the desired effect to occur are not extremely restrictive. Indeed, in the case of steric hindrance, only base pairing is needed, whereas RNase-mediated effects also allow for some relatively important modification of the nucleic acids’ backbone, but 2′ modifications are only allowed in some specific positions in the strand, as the native 2′-deoxy structure (of DNA) is required in the central “gap” region (Figure 4). A comprehensive summary of the medicinal chemistry of the various modifications of ASO and oligonucleotides can be found in a review by Bege and Borbás (2022) [99].

**Figure 6:**
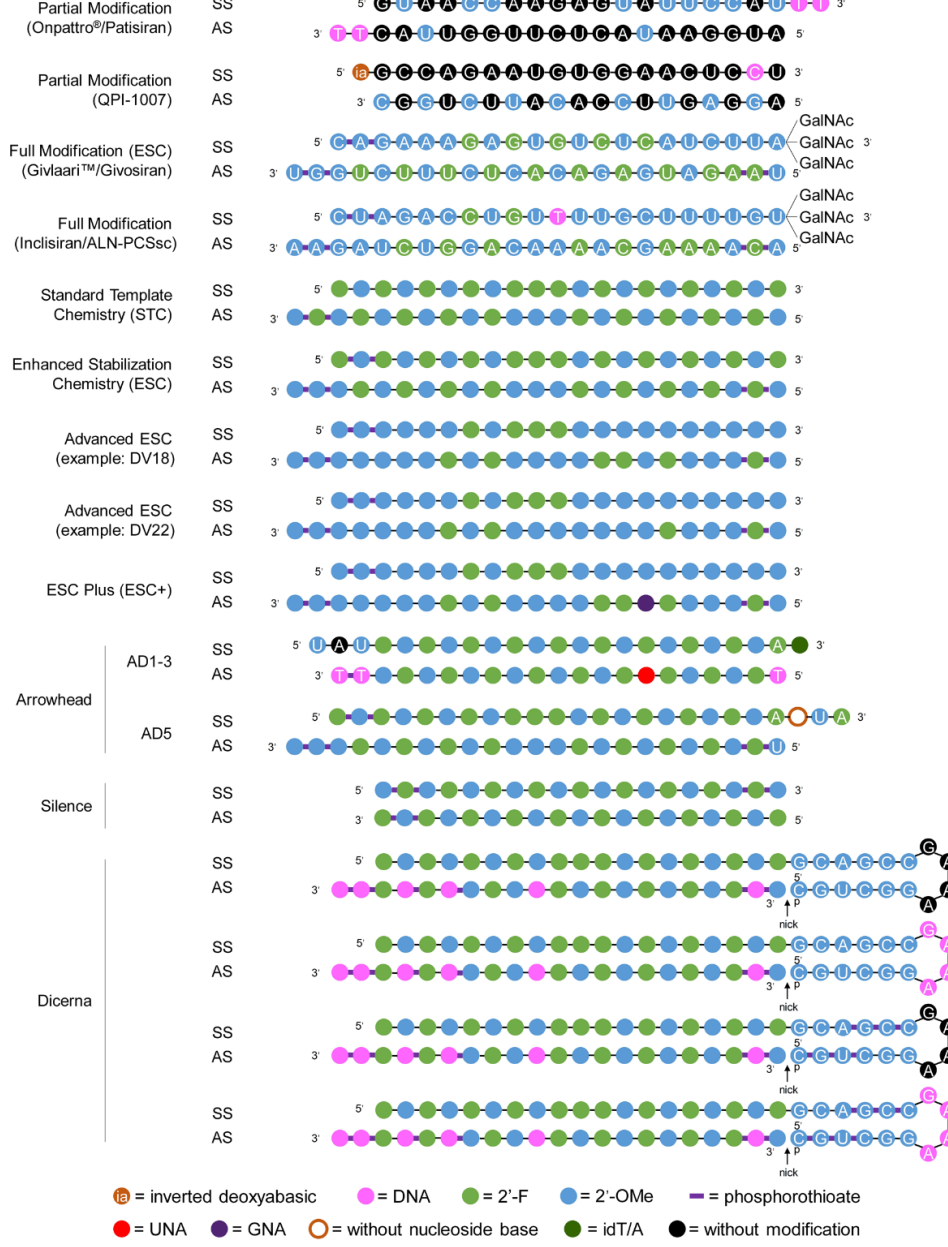
Example of siRNA and shRNA chemical modification platforms. Reproduced with permission from Hu et al., 2020 [32]. In response to the main hurdles faced in nucleic acid therapeutics, one key strategy has been to chemically modify the nucleic acid chains. These modifications are used to enhance stability towards nuclease but also modulate immunogenicity and increase cellular uptake. Most actors in this field have developed their own platforms, using different types of modifications, in different patterns. Interestingly, siRNAs developed by a single company, based on the same platform, show differences in design. This shows that the strategy used in terms of chemical modifications must be adapted on a case-by-case basis to lead to the best compromise between stability, immunogenicity, uptake, and activity.

For siRNAs, however, the backbone cannot be modified as much, whereas the native 2′-OH structure can be quite extensively modified, if it is carried out on specific localizations on the sequence to ensure interaction with the cellular machinery and provide activity. Therefore, different chemical modification strategies are currently explored by the different actors in the field of late-stage clinical siRNAs, leading to different platforms, as illustrated in Figure 6 (taken from Hu et al. (2020) [32]. These platforms incrementally allowed siRNA to have sufficient plasmatic half-lives, from a few minutes to hours in blood, and days to week for elimination in the organism, as well as low immunogenicity and enhanced silencing activity [89,100,101,102,103]. A detailed review by Shukla et al. (2010) [100] also summarizes the different possible modifications and their effect and also proposes a set of rules to design enhanced siRNAs.

In the context of this review, chemical modifications relevant to drug delivery that enabled these nucleic acid modalities to be used in simple formulations in a variety of administration routes will be discussed. As for drug delivery systems and administration routes, we will first look at approved products, then explore how the landscape for chemical modifications is evolving in products in late-stage clinical development.

#### 2.2.1. Approved Products

Table 5 and Table 6 show the chemical modifications observed in commercially available ASOs and siRNAs, respectively. Delivery- or targeting-related ligands are not presented in these tables, as they were discussed earlier.

As can be seen from Table 5 and Table 6, modification of the backbone using phosphorodiamidate morpholino oligomer (PMO) or phosphorothioate (PS) structures is quite common for both modalities, the latter being the most widely used. Indeed, in the case of ASOs, a PS backbone is capable of recruiting RNase H1 for degradation, while having interesting properties in terms of pharmacokinetics and cellular uptake. Furthermore, it also enhances protein binding, which has multiple advantages, such as prolonged plasmatic half-life, which is reported to be up to 2–4 weeks for ASOs with PS-gapmers thanks to nuclease resistance and albumin binding, which reduces renal excretion [96,106]. Another advantage of the protein binding properties of PS-ASOs is the increased interaction with cell surface proteins, which appears to enable internalization and intracellular activity. However, these increased protein interactions with PS-ASOs appear to lead to increased toxicity [107] compared to PMO-ASOs [99]. For siRNAs, however, this backbone modification can unfortunately only be used on the extremities of the strands, as shown in Figure 6; otherwise, the silencing activity is greatly reduced [32].

On the other hand, using a PMO structure on ASOs and siRNAs appears to have a greater effect on the activity. In the case of ASOs, this backbone prevents interaction with RNase H. However, it is especially resistant to degradation and, therefore, appears to be of high interest to design steric-blocking oligonucleotides [108]. For siRNAs though, the use of PMO structures in siRNAs is not possible without impairing the silencing activity. Therefore, the use of this modification is limited to steric-blocking ASOs, which can explain its lower usage. It is, however, worth noting that not all oligonucleotide using that steric-blocking mechanism use a PMO structure, as two such ASOs rely on a more common “PS, 2-O-MOE” format. This last point will be further developed below in Section 2.3, Case Study: Non-RNase H-dependent ASOs. To finish with backbone considerations, one siRNA, Patisiran, uses a standard PO backbone and is the only one that is formulated in a nanocarrier, which seems to underline the importance of such modifications for resistance against nucleases.

No modifications can be seen in the 3′ and 5′ terminal positions except for three of the commercialized PMO-ASOs for DMD, which have a short PEG tail (3 monomers). Although this type of PEG chain can usually be used to prolong the half-life or increase cellular uptake, they are usually of a higher molecular weight [109,110,111]. No definitive explanation could be found regarding the presence of this chain, but one could assume that this is a “leftover” from a solid-phase synthesis step, as described for small batch scale preparations [112], and does not have any particular role in delivery.

Regarding 2′ modifications, despite most siRNAs or ASOs bearing them (considering ASOs that do have available 2′ positions), the type of modification is different between the two modalities. Indeed, it appears that when used in siRNAs, the 2′-O-MOE modification results in lower interference activity [113]; although, it has been recently reported that used in a specific position, in combination with other specific modifications, it could have a beneficial effect on siRNA activity [114]. However, this 2′ moiety is widely used in ASOs, as it is especially efficient to increase binding affinity but most importantly for delivery in reducing toxicity, nuclease sensitivity, and binding to plasma protein, which results in less accumulation of nucleic acids in the liver [115]. This apparent incompatibility between 2′-O-MOE and siRNAs could be an example of how chemical modifications, or the inability to use them in that case, could have an impact on delivery and organ targeting, as contrary to ASOs, the delivery of siRNA is purely liver-centered.

Overall, all the products considered in this section show quite extensive chemical modifications, allowing their use as “free” nucleic acids in biologic media, which would lead to degradation or immune response for native structures.

#### 2.2.2. Late Clinical-Phase Products

Chemical modifications identified in both ASOs and siRNAs in late-stage clinical development are presented in Table 7 and Table 8 below.

Regarding ASOs, it appears that the PS, 2′-O-MOE is still the major structure in use. As for approved products, most of the oligonucleotide bearing these modifications are built as gapmers (Figure 4), while some have all 2′ positions modified and are used as steric blockers. There are, however, two products that use PS, 2′-O-Me and PS, 2′-O-Me, 2′-F modifications for steric blocking. This is especially interesting when put in parallel with the lack of PMO-based steric blockers. As mentioned earlier, the case of non-RNase H-dependent ASOs will be explored in more detail later. Furthermore, it is worth noting that using 2′-F and 2′-O-Me is new for antisense oligonucleotides when comparing to approved products. For siRNAs, on the other hand, there is more variety in the combination of modifications used for molecules in development as compared to approved products, especially when the placement of these modifications along the sequence is considered (Figure 6).

For both modalities, one can, therefore, see that most products in late-stage clinical development are quite extensively modified, with 2′ derivatives and backbone modifications and new inverted bases structures for siRNA. Moreover, there seems to be a strong correlation between the extent of modifications and the use of administration routes that use systemic circulation to reach the target.

On the other hand, more native products, with limited or without 2′ modification or even native backbones, are mostly used for local administration (eye, central nervous system, tumor lesions, GI tract). Regarding ASOs and siRNAs that target the eye (and to some extent the CNS), the lack or low degree of chemical modifications of these products could be linked to different phenomena. Indeed, the eye has lower RNase activity [116] and lowered immune response to extracellular nucleic acids based on the immune privilege of this organ [117]. This could also mean that nucleic acid uptake could be sufficiently quick to protect siRNA from degradation in the cell cytoplasm.

Finally, one product, an IV-administered siRNA that targets the kidney, Teprasiran, is rather interesting in terms of drug delivery. Indeed, this nucleic acid is designed to silence p53 for the treatment of acute kidney injury. In this context, its activity is only required locally, for a short period of time. Therefore, the only enhancement is 2′-O-Me substitution, known to provide reduced immunogenicity, as well as some nuclease resistance [32]. Hence, the biodistribution is not too different from what is to be expected from native siRNA, accumulating in excreting organs, such as the liver and, most importantly, kidney in this case [89], which enables passive kidney accumulation, as discussed above. Moreover, since Teprasiran is not extensively chemically modified, its plasmatic half-life is also rather short, around 20 min. Therefore, this product appears to take good advantage of the properties of siRNAs to reach its target and to have the low duration of action that is desired.

### 2.3. Case Study: Non-RNase H-Dependent ASOs

Of the 28 ASOs considered in this review, 11 are used for their steric-blocking capabilities. Furthermore, of these steric-blocking ASOs, six are used to treat muscle tissues through IV or SC injection, while the others are used for eye or CNS diseases by local injection. This makes for a rather interesting corpus of data for ASO delivery and illustrates more specifically the relationship between chemical structure and delivery, as none of these products use a targeting conjugation, and all are used as simple solutions.

Focusing on the challenges faced for the delivery of ASOs targeting the muscles, the first that can come to mind is that these nucleic acids must be distributed in all muscle tissue. This is a challenge, as muscle tissue is present throughout the body, of which they can account for up to 40% by mass (just for skeletal muscles). This of course makes local injections unfeasible and the use of a systemic route mandatory. Unfortunately, muscles are not typically a site of accumulation for ASOs [41], and their vascularization (accounting for around 20% of total blood flow at rest [118]) is relatively low compared to the tissue mass; although, the variability of the perfusion with physical activity can be quite high.

Considering the different chemical modifications and their properties presented earlier, it appears that two main strategies are available. Indeed, steric-blocking ASOs appear to either rely on a PMO structure or a PS backbone with fully modified 2′ positions. The latter has an increased half-life and is also generally considered to have increased cellular uptake and lower plasmatic clearance compared to native ASOs. PMO-ASOs, on the other hand, while showing excellent resistance towards nucleases, lack negative charges, which can hinder their plasmatic protein binding compared to other oligonucleotide chemistries. This can lead to higher renal clearance and shorter plasmatic half-lives, as well as lowered tissue penetration and cellular uptake compared to other chemistries, such as the previously mentioned PS backbone [119]. Nevertheless, of the six DMD-targeted products identified in this review, four are based on a PMO structure, whereas the two others use a PS backbone with 2′-O-MOE or 2′-O-Me/2′-F modifications. However, only the PMO-ASOs have been FDA-approved, whereas the two PS products’ clinical research was discontinued in late clinical stages, due to a lack of proof of efficacy [120,121]. Furthermore, in a study by Sheng et al. in mice comparing two ASOs based on both PS/2′-O-MOE and PMO chemistries sharing the same sequence, the latter showed higher efficacy in skeletal muscle, which was linked to the increased uptake of PMOs by myocytes [122]. These results could potentially shed some light on the lack of effectiveness that led to the discontinuation of the two PS ASOs. Nevertheless, it is to be noted that in this same study, the PS/2′-O-MOE chemistry displayed more sustained efficacy and overall longer survival.

These elements would seem to imply that the superior cellular uptake of PS-ASOs is not necessarily true for all cell types, or at least not for myocytes. However, it is interesting to note that if these DMD-targeted PMO products have FDA approval, the EMA only appears to have studied one of them, Eteplirsen. This product was refused by the European agency for lack of proof of efficacy, linked to the design of the study but also to the low amount of dystrophin produced [123]. In addition, contrary to the FDA, the EMA seems to rely on functional data (such as walking distance) for drugs to be approved, whereas the FDA accepts surrogate endpoints, such as the level of truncated dystrophins [124]. This could also put in perspective the relative improvement in tissue uptake and the efficacy of the PMO-ASOs discussed above.

Apart from muscles, steric-blocking ASOs (approved or in late clinical-phase trials) target two tissues, the CNS and the eye, all by local administration. Interestingly, none of them rely on a PMO structure, implying that in these cases, the PS-based structures are more advantageous. With clearance being lower in these tissues than in most organs, the preponderance of the PS structures rather than morpholinos seem to imply that in these tissues and target cell types, this type of structure does have an advantage in terms of increased cellular uptake.

Of course, it is important to remember that the approval or discontinuation of these programs cannot be only explained by drug delivery, with multiple factors in play [124]. Nevertheless, these different data seem to support the idea that if systemic delivery of ASO can be obtained, it is not straightforward. Indeed, although for naked ASOs, chemical modifications are mandatory for nuclease resistance, these modifications provide tissue-specific properties in terms of PK, cellular uptake, and overall efficacy. Therefore, when it comes to systemic delivery, it seems that for each oligonucleotide, the target cell type and tissue should be considered in the design of the nucleic acid, along with the desired pharmacological properties. Reciprocally, this also means that the chemistry of ASOs should be considered as an important factor and possibly as a means to achieve a desired delivery, both in terms of targeted tissues and pharmacokinetics profile.

### 2.4. Conclusion and Perspective on Antisense Oligonucleotide and siRNAs Delivery

Based on these elements, it appears that thanks to chemical modification strategies, smaller nucleic acids modalities, such as ASOs and siRNAs, are now at a point where stability and toxicity are somewhat controlled and compatible with unencapsulated therapeutic use. These chemical modifications also appear to allow for sufficient cellular uptake in local administration, especially for ASOs for which there are rather interesting administration routes, such as delayed release tablets [125], eyedrops, or enemas. Many more different chemical modifications are also described in the literature, such as locked nucleic acids or peptide nucleic acids, for example [99,126], which might also further enhance the potential of these nucleic acids for systemic and local administration. However, it appears that there are fewer target organs and administration routes for siRNAs compared to ASOs. This difference might stem from multiple factors, such as the higher molecular weight, relative fragility, and overall strong hepatic tropism of these double-stranded species. These elements could to some extent be linked to the lower tolerance of siRNAs towards chemical modifications, which have been shown to play a major role in many drug delivery aspects. Overall, the further exploration of the local delivery of ASOs and especially siRNAs appears to be an interesting and important path to extend the list of attainable organs and associated diseases that can be treated.

Furthermore, based on the low representation of systemic treatments, strategies to deliver treatments to specific organs systemically appear as a major unmet need. Indeed, for the moment, the delivery of siRNAs and ASOs is limited to tissues for which topical administration is readily possible or to tissues that have specific properties facilitating systemic delivery (whether it is natural tropism or cellular receptors facilitating active targeting). The development of advanced DDS could, therefore, enable more tissues to be reached or make targeting of certain organs simpler, facilitating access to such therapies and improving compliance by enabling simple infusion methods (one could argue that if it was possible, replacing a local intraocular injection by a subcutaneous injection of a DDS targeting the eye, for example, would most likely be preferred by the patients).

Nevertheless, despite obviously complicating development, manufacturing as well as regulatory and quality aspects of nucleic acid products, such DDS would most likely require more than a simple conjugation to a targeting moiety to show sufficient efficacy. Indeed, there is a tremendous lack of variety in active targeting moieties in the products reviewed here, compared to the extensive lists of targeting agents available in the literature [127,128]. This discrepancy highlights the fact that targeting a nucleic acid towards an extracellular receptor is not sufficient to ensure efficient cellular uptake and efficacy [45,129].

This is especially striking when considering the major precedent that the GalNac moiety provided by “solving” hepatocyte delivery. After this strategy proved to have such a tremendous impact, one could imagine that many other conjugates would have been tested, and that some of them would have reached late clinical stage. However, as stated earlier, the ASGPR receptor has incredibly high cellular uptake capabilities that are not usually found in other tissues. Nonetheless, this high cellular uptake does not actually solve the main limiting factor to nucleic acids’ efficacy but rather compensates for the true barrier for nucleic acids efficacy—endosomal escape [45,50,51]. It seems like this previous observation can be extended to all siRNAs and ASOs studied here. Indeed, whether a local injection is used to reach a high local concentration, or a conjugate is made to increase cellular uptake, the objective remains to compensate for the low proportion of therapeutic agent that reaches the cytoplasm.

As these products are designed to treat chronic diseases, an important point to consider is the posology of nucleic acid-based therapies. Looking at approved ASOs, two main scenarios seem to emerge with respect to posology, depending on the route of administration, as follows: for local products low doses are found, from less than a mg (intraocular) to a few tens of mg (intrathecal), whereas for systemic routes targeting muscles or liver, they are in the range of 200 mg to more than 2000 mg. In terms of posology, it appears that intrathecal products require injections every few months, while on the other hand, for systemic and intraocular products, injections are carried out weekly to biweekly. Therefore, it looks like there might be an interest for sustained release formulation of ASOs for local injections in some specific cases. Indeed, for intrathecal products, although the route is rather invasive, the delay between injections seems acceptable and compatible with a traditional follow-up appointments schedule with a specialist. For liver- or muscle-targeted products, if the shorter delay between injections looks like it could benefit from sustained release formulations, the relatively high doses might make it difficult to translate the idea into monthly and especially bimonthly or quarterly products. For intraocular products though, the delay between injection is relatively short, doses are low, and the route is quite invasive, making it a rather good candidate for sustained release formulations.

Finally, regarding siRNAs, it seems like sustained release formulations might not appear as a priority in the current state of their delivery. Indeed, for GalNac conjugates, intervals between administrations go from one to six months with subcutaneous injections. For Patisiran, the only LNP-formulated siRNA on this list, the time between injections remains relatively long; although, it is shorter and maybe more inconvenient for patients than other products, with IV injection every three weeks. This may mean that for encapsulated siRNA, sustained release formulations, e.g., a reservoir system releasing nanoparticles over time, could be of interest. Nevertheless, for the moment, the encapsulation of these nucleic acids is not really represented in either marketed products or in molecules in late-stage clinical development. On the other hand, going back to the need for targeted systemic treatments, this type of DDS could most likely use particle vectors to deliver siRNA in the future, in which case a sustained delivery formulation could complete the drug delivery system. It is also important to note that if new local administration strategies are used in the future, like for ASOs, sustained release formulations might also become interesting.

## 3. mRNA

### 3.1. Challenges Compared to Other Nucleic Acids

mRNAs, which can turn human cells into endogenous protein production units, are the youngest representative of the therapeutic nucleic acid modalities on the market. Indeed, the first two and only approved products (at the time of writing this review) are COVID-19 vaccines, which received their approval in 2021. This later adoption of mRNAs in the therapeutic arsenal may find its root in the relative difficulty to turn these nuclease-sensitive, immunogenic, high-molecular-weight, intracellularly active molecules into drug products.

Indeed, the mean molecular weight of nucleotides is around 330 Da, coding in a triplet for a single amino acid whose mean molecular weight is around 110 Da. This means that the molecular weight of just the coding sequence of an mRNA will be ten-fold higher than the protein it codes for [17]. Pfizer-BioNTech’s vaccine, for example, uses a 4284 base nucleic acid sequence [130], therefore corresponding to roughly 1.4 MDa, bearing more than four thousand negative charges, as all phosphates of the backbone are expected to be charged at physiological pH [131]. Besides mRNAs’ less than ideal physicochemical characteristics for membrane permeation, the stability of such nucleic acids is also a problem. Indeed, as with all polymers, a longer chain usually increases the probability of cleavage. Moreover, the main instability is enzymatic due to cleavage by ubiquitous nucleases and RNases [132], as for other nucleic acids. Finally, as evoked for other modalities, mRNA can be immunogenic, activating different innate immune response pathways [133,134]. It is also to be noted that double-stranded RNA, which can be a by-product of in vitro synthesis, can be seen as a danger signal by the immune system [135,136].

Unfortunately, as opposed to ASOs or siRNAs, the leeway regarding chemical modifications is much narrower for mRNAs. Indeed, if many modifications in the 5′ and 3′ untranslated regions (UTRs), such as caps, PolyA tails, and even PS backbone, can be seen in the literature [137,138], data regarding open reading frame (ORF)-modified mRNAs are rather scarce. This could be explained by the complex effects that modifications in this region can have on mRNA translation [139] and the subsequent activity of the treatment. Most importantly, however, this seems to restrict and condition the possibilities in terms of formulation and delivery, as mRNA delivery appears to be stuck in a paradigm of forced encapsulation to limit degradation and immune response.

### 3.2. mRNA Delivery

Overall, the previous elements tend to indicate that for the moment mRNA delivery is bound to rely on carriers to encapsulate these nucleic acids and provide them with the required pharmacokinetic properties, as well as cellular uptake capabilities, and lowered immunogenicity. Table 9 and Table 10 summarize delivery-relevant data in approved and late-development mRNA-based products.

As expected, most mRNA products use a carrier as the drug delivery system, and the majority of carriers are lipid nanoparticles (LNP), with only one lipopolyplex, and two naked mRNA transfected by electroporation into dendritic cells ex vivo. Regarding the route of administration and target, not much can be said for mRNA. Indeed, not counting for the transfected dendritic cells, 19 out of the 22 remaining products are for intramuscular injections, whereas for the rest, it is undisclosed.

At this point, it is important to add that all products are vaccines, which ties together the common choice of injection route and drug delivery system. Indeed, it is common for vaccines to be injected in muscles, because this tissue has a good vascularization, which provides good drainage towards lymphatic nodes and a good population of immune cells, such as dendritic cells (DC) [170]. In addition, the choice of LNP formulations is advantageous in terms of both immunostimulation and delivery [171,172]. Indeed, LNPs have an important proportion of ionizable or cationic lipids, which can have stimulatory effect on the immune system and recruit antigen presenting cells (APC) [173] but also facilitate cellular uptake and endosomal escape [137]. Interestingly, one of these products uses a lipopolyplex DDS. These nanoparticles are composed of a polyplex core, which is a complex of cationic polymers and RNA, and a lipidic corona. This formulation is said to give good immune cell stimulation thanks to the lipidic corona, while providing good encapsulation and the progressive release of the mRNA in cells linked to the polymeric core [174].

Finally, the case of the CureVac CVnCOV vaccine is interesting, as its clinical development has been discontinued, apparently due to low efficacy [175]. One can see that amongst the mRNA vaccines discussed here, it is one of the few that using unmodified nucleotide. This is most likely the reason that led to the use of much lower amounts of mRNA per vaccination shot, as unmodified ribonucleotide can be quite immunogenic and lead to side effects. These lower amounts of mRNA may have led to a lower amount of expressed spike protein and thus lower immunization [176]. This is, therefore, a good illustration that if the delivery systems are key for mRNA delivery, just like siRNAs or ASOs, modifications of the chemical structure of the sequences are of paramount importance, but also that development of an mRNA vaccine is a balancing act. Indeed, like many other vaccines using adjuvants, immunogenicity is required to some extent to provide vaccination. The immune reaction should, however, be directed towards the desired antigen, in a controlled magnitude. This is where the balance between the modification of the mRNAs and the use of immunogenic DDS must be carefully observed to provide the desired vaccination.

### 3.3. Conclusion and Perspective on mRNA Delivery

The field of late-stage clinical or marketed mRNA products stands out by its lack of diversity. Of course, the COVID-19 outbreak had an important role in this situation; as there was a global demand for a vaccine, mRNA-LNP vaccines appeared as an ideal solution. This sparked a wave of interest in the field, and soon, many vaccine trials were started using this platform. If this was highly beneficial in bringing more light to the many applications of RNAs, especially on the industrial side of things [30,177,178], it did not bring a lot of variety, as the urgency of the situation led to a clear preference for the use of tried and proven concepts. However, there are most likely other reasons for the over representation of vaccine products and LNPs.

First, if LNPs are described as good DDS for mRNA, one must keep in mind that their performance is relative to other technologies. Indeed, the efficiency of translation appears to be rather low, mostly linked to the poor endosomal escape of the nanoparticles leading to degradation in the lysozyme before reaching the cytoplasm [45,179]. If for vaccines a small amount of protein expressed during a rather short period of time can be sufficient to elicit a sufficient immune response, for other application, such as protein supplementation, the aforementioned characteristics of LNP DDS can become limiting factors [133,134,180].

Furthermore, in the context of protein replacement therapy, other problems with the use of LNPs arise. The most obvious is probably the tropism of LNPs for the liver, as described earlier [181]. This inherent tropism is one additional barrier to bring an mRNA in a desired organ at a sufficient concentration. Furthermore, as stated earlier, the protein expression observed after the IM injection of an mRNA vaccine is transient. This means that repeated injections must be used in the case of chronic diseases for which protein replacement therapies are required. From this arises the question of toxicity after repeated injection, whether it is toxicity from mRNA itself [133], cationic or ionizable lipids [182,183], or to the PEG corona that is typically used to stabilize LNPs [184,185]. It is important to note that in parallel to new delivery strategies, newer RNA modalities, such as saRNAs, which could in theory yield higher and sustained protein expression levels [186], might play an important role in leveraging RNA-based protein replacement therapies.

Finally, as the COVID-19 pandemic clearly illustrated, the handling and conservation of mRNA products is currently a logistical challenge. Indeed, current LNP formulation required storage at −20 °C to −80 °C with rather limited shelf lives [129,130]. This stability issue stems not only from the mRNA itself but also from the nanoparticles whose properties can be altered by drying or improper freeze/thaw cycles. This limitation is especially important in the context of vaccines whose activity relies on the broad immunization of the population, which is a logistical challenge, even without accounting for demanding storage conditions.

Of course, the world of mRNA (and gene delivery in general) does not stop with LNPs, as many other technologies are described in the literature, such as polymeric nanoparticles, extracellular vesicles (EVs), adenoviruses, and peptide-based nanoparticles [137,174,187]. Each of these technologies has its own advantages and disadvantages and should, therefore, be considered in light of the target organ or cell and therapeutic effect. Although one could argue that from an industrial point of view, harnessing the full potential of these technologies may be more difficult compared to using LNPs. Indeed, to formulate LNPs, one would essentially need access to the subcomponent of the system (the lipids, many of them are commercially available) and process knowledge. If this is complex enough, screening many different LNP formulation is relatively easy, as it usually consists of testing different compositions with different process parameters.

On the other hand, the development of polymer-based DDS (polyplex), for example, can be more complicated in an industrial setting than LNPs. Indeed, screening different formulations of polyplex DDS for optimized delivery can imply testing many polymer modifications, such as molecular weight, chain structure, substituents, and substitution ratios, not all of which will be commercially available. Thus, full synthesis of new polymers could be needed, with all the specialized knowledge needed to have control over (and monitor) quality attributes. This polymer expertise can be complicated for pharmaceutical companies that are not specialized in this type of synthesis to acquire and develop and can, therefore, be an obstacle to polyplex use. In comparison, for lipid-based nanoparticles, many lipids are commercially available, allowing a laboratory to more easily screen many more formulations, playing on composition ratios and many process parameters.

Another example of DDS that despite having great potential are not preferred to LNPs are extracellular vesicles or adenoviruses. Indeed, if usually described as efficient DDS, they are biological products and, therefore, tend to be more complicated to develop, produce, and characterize. It is also important to note that one of the key advantages of mRNA is their in vitro production, which is performed in the absence of cells and, therefore, avoids virus or cell fragment contamination problems. Using DDS, such as viruses or EVs, could, therefore, bring back a concern that RNA technology allows us to avoid.

Overall, the next steps of mRNA delivery research appear to be clear. To harness the full potential of this nucleic acid modality outside of its use for vaccines, the focus should be put on enhancing endosomal escape of the nanoparticles, which is considered to be the main bottleneck for mRNA efficacy [187], targeting the therapies to desired tissues other than the liver, and finally, the stability of the drug products. However, it appears that a holistic approach should be used with regard to endosomal escape and targeting aspects for each application. Indeed, depending on the DDS and targeting modality chosen to reach a specific cell type, internalization pathways and cellular environment may be greatly different, as illustrated in Figure 5. It must be noted that addressing these problematics, more specifically endosomal escape and targeting, might lead to more complex DDS. Thus, it could be expected that new challenges could arise in the future, mostly CMC-related, such as downstream processes and the characterization of these formulations.

## 4. Aptamers

Aptamers are rather short, single-stranded nucleic acid-based molecules, usually in the range of 10 to 30 kDa (30 to 100 nucleotides). These nucleic acids have the particularity of forming distinct 3D structures through internal base pairing, giving them specific recognition and binding properties, much like antibodies (around 150 kDa) [188]. Aptamers have multiple advantages over antibodies, such as their smaller size (giving them potentially more interesting tissues penetration properties than larger antibodies [189]), their lower immunogenicity, or the many type of target they are able to bind to (peptides, nucleic acids, metal ions…) [190]. Aptamers also have advantages over antibodies in terms of development and production, as these steps can be conducted entirely in vitro in a cell-free manner [191]. Nevertheless, on the other hand, their nucleic acid structure makes them susceptible to nuclease-based degradation and resulting issues with respect to their pharmacokinetics. These molecules have, therefore, been used in many different ways, either as therapeutics, as part of targeted drug delivery systems [16], or as powerful research tools [190]. In this review, the focus will be on their use as therapeutic agents.

### 4.1. Structure-Based Approach to Delivery

The strategies used to deliver aptamers are very similar to those used for other smaller nucleic acid modalities seen in this review. Indeed, in a first step, nucleic acids are made more drug-like, using a set of different chemical modifications. These modifications aim at giving these aptamers better immunological profiles, nuclease resistance, and improved pharmacokinetic properties.

Table 11 and Table 12 illustrate the chemical modification observed for aptamers that are approved or in the late clinical phase (Phase 2/3 and 3).

Interestingly, one can see that none of the products use the modified backbone chemistry seen above; although, it is known to promote better nuclease resistance and is a modification that is described for aptamer in the literature. The toxicity of fully PS-modified backbone, for example, may be a reason for that [197]. Furthermore, as all these aptamers have extracellular targets, one could hypothesize that the increased protein binding and cellular uptake observed with this modification might hinder in vivo activity. Therefore, it appears that the modification strategy here is different to that for siRNA and ASOs. Indeed, an increase in half-life (which in this case encompasses RNase degradation and clearance) seems to rely for most aptamers on 2′ modification and PEG conjugation; 2′ modifications are present on all products for which data are available and thus appear to be relatively efficient on their own for RNase resistance. In the case of Pegpleranib, for example, a 13-fold increase in plasma half-life was observed for the fully modified aptamer compared to its DNA or RNA parent.

Another important point is the omnipresence of PEG conjugation. These polymeric chains are usually rather long (two 20 kDa chains for Pegaptanib [198]) and are mainly used to modify aptamer size, hence modifying their pharmacokinetics. For example, when combined with nuclease resistance-improving modifications, the PEGylation of Pegaptanib led to an increase in the plasma half-life of this aptamer from a few minutes to more than nine hours [199]. Another interesting use of polyethylene chains is illustrated with Pegpleranib, in which some nucleotide loops in the parent aptamer sequence were replaced with short PEG chains. This led to presumably better endonuclease resistance by removing enzymatic substrate sites wherever possible, as well as simpler (and thus cheaper) synthesis by reducing the number of elements to link in the chain [200].

Finally, one product from this list, Defibrotide, has a rather particular profile, as it is a complex mixture of oligonucleotides extracted from animal tissues. If the exact mechanism of action of this product has not been completely elucidated yet, it is an antithrombotic used for severe hepatic veno-occlusive disease. It is, however, known that it contains aptamers binding thrombin, which is why it is considered here [201]. Due to the nature of this product, it is difficult to detail its chemical modifications; although, it is expected that they would have at least some common modifications found in endogenous nucleic acids [202,203]. Nevertheless, these nucleic acids appear to have a relatively short half-life, shorter than an hour [204]. Although it does not seem like the structures of the nucleic acids are made by design, this product appears to be a good example of how the native properties of aptamers, such as short half-life and liver tropism, can be used as an advantage for delivery.

### 4.2. Delivery of Aptamers

As seen above, aptamers, like siRNA or ASOs, have relatively drug-like properties in terms of pharmacokinetics.

Table 13 and Table 14 show the target organs and corresponding administration route used for aptamers.

Interestingly, for aptamers, only two target organs are presented here—the eye and the blood–more specifically, the different agent responsible for hemostasis. Moreover, the molecular targets are all soluble, extracellular proteins (not accounting for the unelucidated targets of defibrotide). This could explain why no DDS is used for aptamers, which rely on local administration for each product (in this case IV injection can be regarded as “local” injection for products like Defibrotide or Pegnivacogin, which have antithrombotic activities. For Defibrotide, it is also interesting to note that its indication is hepatic veno-occlusive disease. As for oligonucleotides seen in this review, liver biodistribution of aptamers is relatively high, most likely due to its filtering role [205]. Therefore, it appears that this product without conjugation or DDS already has relatively good properties for its intended use. The high representation of eye targets can also be explained by favorable properties of this modality in this particular tissue, such as good tolerability, residence time, etc. [116].

### 4.3. Conclusion and Perspective on Aptamer Delivery

Overall, it appears that aptamers are in a relatively similar place to ASOs and siRNAs in terms of drug delivery. Indeed, thanks to the leeway regarding structure and chemical modifications in these products, nuclease resistance and renal filtration can be controlled, leading to increased half-life in tissues, effectively modifying tissue distribution and delivery. It seems that fewer chemical modifications have been explored in late clinical-phase trials for aptamers in comparison, such as PS backbones or 2′-O-MOE.

In addition, a key difference with other modalities is that the aptamer targets do not necessarily have to be intracellular. Therefore, it seems that less effort has been made in terms of conjugations or DDS to increase cellular uptake. Nevertheless, development of such intracellular-targeted aptamers could be of high interest [206], as it is one the advantages that they could have over antibodies, with which they are often compared [191]. The variety of targets is also quite poor with this modality, with the only targets being plasmatic or ocular.

Regarding dose and posology, the two approved products have rather opposite profiles. For Defibrotide, the dose is rather high at 6.25 mg/kg (around 400 mg for a 70 kg patient) every 6 h by intravascular injection, whereas the Pegaptanib dose is in hundreds of micrograms, every 6 months. In both cases, sustained release formulations appear unapplicable, in the first case due to the high daily dose, and in the second one because the product itself already has rather long interval between administrations.

The diversification of the chemical modifications might further increase the pharmacokinetic profile of aptamer and could also increase cellular uptake [107], offering more potential targets. The future of aptamer delivery should also focus on expanding the target organs, whether it is by diversification of the local administration routes or with more advanced DDS, such as conjugates or targeted particles.

## 5. Conclusions

In this review, the focus has been put on approved RNA products and molecules that have reached late clinical-phase trials, since this can be considered as a gauge of success in overcoming the different challenges faced during the development of RNA therapeutics. Although we have looked at the four main classes based on approved products, other classes of nucleic acids are also currently in clinical phases, e.g., gRNA (guide RNA), saRNA (self-amplifying RNA), shRNA (short hairpin RNA), or various oligonucleotides. These other classes have intentionally not been treated here, because of the small number of molecules concerned in each class, making it difficult to extrapolate trends and identify-specific therapeutic or technical needs. The delivery-related data corresponding to these products are, however, available in the Appendix A (Table A1).

Nevertheless, more generally speaking for RNA-based products, the current situation in terms of drug delivery is rather interesting. Indeed, thanks to years of research and the recent explosion of interest in the field, there is far more knowledge and understanding of their mechanism of action in relation to their structure. This has led to these chemical entities, whose main drawback is their fragility, becoming more drug-like, accentuating their potential advantages as therapeutic agents. Nevertheless, as compared to products that are more advanced in their life cycle, the variety of drug delivery technologies applied, and thus the efficiency in targeting tissues, is rather low—except perhaps for the liver. Hence, ribonucleic acids appear to be in a place where they are mature as therapeutic agents and could benefit from applying drug delivery technologies.

Regarding what is currently carried out in terms of DDS, it looks like two main scenarios can be observed, depending on whether the nucleic acids are short or long. For longer nucleic acids, such as mRNAs, it appears that the limitations in terms of chemical modifications and their size make it mandatory to use rather elaborate DDS, such as nanoparticles or physical transfection methods, like electroporation. If the first option enables more traditional administration strategies (i.e., injections), it appears that the properties of the carriers can make it difficult to balance tissue targeting, efficacy, good tolerability, manufacture, characterization, and storage. This results in a small number of applications, which mainly benefit from the tropism of a DDS towards specific tissues presenting particular physiological properties. Thankfully, a wide variety of DDS are described in the literature, which should hopefully enter more advanced development phases and allow a wider range of targets to be reached in the future. On the other hand, if ex vivo strategies, such as electroporation, allow for cell-specific, highly efficient transfection, the logistics and highly specialized knowledge and equipment needed may hinder their use as therapeutics for most patients. For smaller nucleic acids, such as ASOs, siRNAs, or even aptamers, their intrinsic properties and chemical modifications make them usable without carriers allowing for greater diversity in their targets and DDS. With these modalities, many different local administration strategies could be investigated, as well as conjugate strategies, whilst using mostly “naked” nucleic acids.

Now, it is important to emphasize the limiting factor with all these modalities, which is the endosomal escape after cellular uptake, since endocytosis is the main path for cellular uptake. However, as different tissues and cellular types may display different internalization pathways, it is important to consider the delivery of nucleic acid in a holistic approach, taking into account the target tissues or cells, the DDS, and the payload.

Overall, the main focus of further development in the delivery of the nucleic acids’ modalities discussed in this review should be on the endosomal escape/targeting of therapeutics, which should be considered as a single problem for each therapeutic and could be addressed in many different ways, such as exploring different nanocarriers for larger nucleic acids or different conjugations and local administration strategies for smaller entities. It is important to note that many other modalities currently in earlier stages of development, like circular RNAs, may bring new opportunities in therapeutics and delivery, as well as new challenges.

## Figures and Tables

**Table 1:** Approved ASOs and their delivery characteristics.

INN Name	Approval(s)	Target Site (Target)	Disease	Posology	Delivery Route	Delivery System	Conjugation/Termination
Casimersen (Amondys 45)	FDA 2021	Muscle fibers (DMD exon 45)	Duchenne muscular dystrophy (DMD)	30 mg/kg q.w.	Intravenous (IV)	Naked in PBS	Short PEG tail
Eteplirsen (Exondys 51)	FDA 2016	Muscle fibers (DMD exon 51)	Duchenne muscular dystrophy	30 mg/kg q.w.	IV	Naked in ~PBS	Short PEG tail
Golodirsen (Vyondys 53)	FDA 2019	Muscle fibers (DMD exon 53)	Duchenne muscular dystrophy	30 mg/kg q.w.	IV	Naked in PBS	Short PEG tail
Milasen^117^	FDA 2017	SNC (CLN7)	Batten’s disease (patient-customized)	42 mg quarterly after induction phase	Intrathecal (ITh)	Undisclosed	NA
Nusinersen (Spinraza)	FDA 2016 EMA 2017	SNC (SMN2 pre-mRNA)	Spinal muscular atrophy	12 mg every four months after induction phase	ITh	Naked in ~PBS	NA
Viltolarsen^118^ (Viltepso)	FDA 2020	Muscle fibers (DMD exon 53)	Duchenne muscular dystrophy	80 mg/kg q.w.	IV	Naked in pH-adjusted saline	NA
Fomivirsen (Vitravene)	FDA 1998 EMA 1999	Eye (CMV mRNA)	Cytomegalovirus retinitis	0.165/0.330 mg/eye q.2w after induction phase	Intravitreal (IVt)	Naked in bicarbonate buffer, NaCl	NA
Inotersen (Tegsedi)	FDA 2018 EMA 2018	Liver (TTR mRNA)	Hereditary transthyretin mediated amyloidosis	285 mg q.w.	Sub- cutaneous (SC)	Naked in pH-adjusted water	NA
Mipomersen (Kynamro)	FDA 2013	Liver/Intestine (apo-B-100 mRNA)	Familial hypercholesterolemia	200 mg q.w.	SC	Naked in pH-adjusted water	NA
Volanesorsen (Waylivra)	EMA 2019	Liver/Intestine (apoC-III mRNA)	Familial chylomicronemia	285 mg q.2w after induction phase	SC	Naked in pH-adjusted water	NA

**Table 2:** Approved siRNAs and their delivery relevant characteristics.

INN Name	Approval(s)	Target Site (Target)	Disease	Posology	Delivery Route	Delivery System	Conjugation/Termination
Givosiran (Givlaari) [35]	FDA 2019 EMA 2020	Liver/hepato-cytes (ALS1 mRNA)	Acute hepatic porphyria	2.5 mg/kg q.m.	SC	Naked in pH-adjusted water	GalNAc-conjugate
Inclisiran (Leqvio) [32,36]	EMA 2020	Liver/hepatocytes (PCSK9 mRNA)	Primary hypercholesterolemia	284 mg every six months after induction phase	SC	Naked in pH-adjusted water	GalNAc-conjugate
Lumasiran (Oxlumo) [37]	FDA 2020 EMA 2020	Liver/hepatocytes (HAO1 mRNA)	Primary hyperoxaluria	3 to 6 mg/kg quarterly after induction phase	SC	Naked in pH-adjusted water	GalNAc-conjugate
Vutrisiran (Amvuttra) [38,39,40]	FDA 2022 EMA 2022	Liver/hepato-cytes (TTR mRNA)	hATTR amyloidosis	25 mg quarterly	SC	Naked in phosphate buffer + NaCl	GalNAc-conjugate
Patisiran (Onpattro)	FDA 2018 EMA 2018	Liver (TTR mRNA)	hATTR amyloidosis	0.3 mg/kg every three weeks	IV	Lipid nanoparticles (LNPs)	NA

**Table 3:** ASOs in the late clinical phase and their delivery characteristics.

Drug Names	Clinical Trial Status	Target Site (Target)	Disease Indication	Deliv. Route	Deliv. System	Conjugation/Termination
Tofersen */BIIB067/IONIS-SOD1Rx/ISIS 333611/BIIB067/IONIS-SOD1Rx/ISIS 333611 [52]	Ongoing	CNS (SOD1 mRNA)	Amyotrophic lateral sclerosis (ALS); superoxide dismutase 1-amyotropic lateral sclerosis (SOD1-ALS)	ITh	Naked RNA	NA
Tominersen/IONIS-HTTRx/ISIS 443139/RG6042/RO7234292 [52,53]	Ongoing	CNS (HTT mRNA)	Huntington’s disease	ITh	Naked RNA	NA
Zilganersen/ION-1166998/ION373 [54]	Ongoing	CNS (GFAP mRNA)	Alexander disease	ITh	Undisclosed	NA
Sepofarsen/ QR-110 [55]	Ongoing	Eye (CEP290 mRNA)	Blindness; congenital eye disorders; eye disorders; Leber congenital amaurosis (LCA); Leber congenital amaurosis 10 (LCA10); neurological disorders; retinal degeneration; retinal disease; retinal dystrophy; sensation disorders; vision disorders	Intraocular (IO)	Naked RNA	NA
OT-101/AP 12009/AP-12009/ATB-301/TASO-001/TGF-β2 targeting anti-sense oligonucleotide/Trabedersen [56]	Ongoing	Tumor (TGF-B2 mRNA)	Advanced malignant pleural mesothelioma; advanced solid tumors; cancer indications; colorectal cancer; diffuse intrinsic pontine glioma; glioma; melanoma; metastatic solid tumors; pancreatic cancer; pleural malignant mesothelioma; progressive myopia; SARS-CoV-2 (COVID-19) infection; solid tumors	Intratumoral (IT)	Undisclosed	NA
Drisapersen/GSK2402968/Kyndrisa/PRO051 [57,58]	Stopped	Muscles (DMD exon 51)	Duchenne muscular dystrophy (DMD); muscular dystrophies	SC	Undisclosed	NA
Ultevursen/QR-421a [59,60]	Ongoing	Eye (USH2A exon 13)	Blindness; congenital eye disorders; deafness; eye disorders; hereditary conditions; retinal disease; retinitis pigmentosa; Usher syndrome type 2; Usher syndrome type 2a; vision disorders	IO	Undisclosed	NA
Bepirovirsen/GSK3228836/IONIS-HBVRx/ISIS 505358/ISIS-GSK3Rx/ISIS-HBVRx [61]	Ongoing	Liver (HBsAg mRNA)	Hepatic impairment; hepatitis B; liver cirrhosis; liver disorders	SC	Undisclosed	NA
Mongersen/GED-0301 [62]	Ongoing	GI epithelium (SMAD7 mRNA)	Crohn’s disease; ulcerative colitis	Oral	Delayed release tablet	NA
IONIS-PKK-LRx/Donidalorsen/ISIS 721744 [63,64]	Ongoing	Liver (KLKB1 mRNA)	Cardiovascular diseases; hereditary angioedema; SARS-CoV-2 (COVID-19) infection	SC	Undisclosed	Ligand-conjugated antisense (LICA) (GalNac)
Eplontersen */AKCEA-TTR-LRx/ION-682884/IONIS-TTR-LRx/ISIS-TTR(Rx) [65,66]	Ongoing	Liver (TTR mRNA)	Amyloidosis; familial amyloid polyneuropathy (hereditary transthyretin-mediated amyloid polyneuropathy); transthyretin amyloid cardiomyopathy	SC	Undisclosed	Ligand-conjugated antisense (LICA) (GalNac)
ION363/Jacifusen [67]	Ongoing	CNS (FUS mRNA)	Amyotrophic lateral sclerosis (ALS)	ITh	AAV (Adeno Associated Virus)	NA
Custirsen/OGX-011/TV-1011 [68,69]	Not active (clinical)	Tumor (sCLU mRNA)	Adenocarcinoma of the prostate; advanced non-small cell lung cancer (NSCLC); cancer indications; cardiac conduction and repolarization; castrate prostate cancer; metastatic castrate prostate cancer; metastatic non-small cell lung cancer; prostate cancer; solid tumors	IV	Undisclosed	NA
Pelacarsen/AKCEA-APO(a)-LRx/ASO 144367/IONIS-APO(a)-LRx/ISIS 681257/TQJ230 [70]	Ongoing	Liver (LPA mRNA)	Acute coronary syndrome; cardiovascular diseases; coronary artery disease (coronary heart disease); elevated serum lipoprotein(a); genetic polymorphisms; hepatic impairment; hyperlipoproteinemia (hyperlipidemia, lipoproteinemia); inflammation; ischemic stroke; liver cirrhosis; myocardial infarction; peripheral arterial disease (PAD); renal impairment; stenosis	SC	Undisclosed	Ligand-Conjugated Antisense (LICA) (GalNac)
Suvodirsen/WVE-210201 [71]	Stopped	Muscles (DMD mRNA)	Duchenne muscular dystrophy (DMD)	IV	Undisclosed	NA
Aganirsen/GS-101 [72,73]	Ongoing	Eye (IRS1 mRNA)	Ischemic central retinal vein occlusion; neovascular glaucoma	Eye, Topical	Naked RNA	NA
Alicaforsen/ISIS-2302 [74]	Ongoing	Colon (undisclosed)	Inflammatory bowel disease; pouchitis; ulcerative colitis	Rectal	Naked RNA	NA
Olezarsen */AKCEA-APOCIII-LRx/IONIS APOCIII-LRx/ISIS 678354 [75,76,77]	Ongoing	Liver (APOC3 mRNA)	Atherosclerosis; atherosclerotic cardiovascular disease (ASCVD); cardiovascular diseases; coronary artery disease (coronary heart disease); elevated triglycerides; familial chylomicronemia syndrome (FCS); familial lipoprotein lipase deficiency (hyperlipoproteinemia type I); hypertriglyceridemia; ischemic stroke; pancreatitis; peripheral arterial disease (PAD)	SC	Undisclosed	Ligand-conjugated antisense (LICA) (GalNac)

*: These products have been approved since the data for this review were collected.

**Table 4:** siRNAs in the late clinical phase and their delivery characteristics.

Drug Names	Clinical Trial Status	Target Site (Target)	Disease Indication	Deliv. Route	Deliv. System	Conjugation/Termination
Revusiran/ALN-TTRsc/SAR438714/siTTRsc [78]	Stopped	Liver (TTR mRNA)	Amyloidosis; cardiac amyloidosis; familial amyloid polyneuropathy (hereditary transthyretin-mediated amyloid polyneuropathy); hereditary ATTR amyloidosis	SC	Undisclosed	GalNAc
Teprasiran/I5NP/QPI-1002 [79]	Ongoing	Kidney (p53 mRNA)	Acute kidney injury (AKI); renal failure	IV	Undisclosed	NA
Tivanisiran/SYL1001 [80]	Ongoing	Eye (TRPV1 mRNA)	Dry eye disease; ocular Pain; Sjögren’s syndrome	Eye, topical	Naked RNA	NA
Nedosiran */DCR-PHXC [81]	Ongoing	Liver (LDHA mRNA)	Genetic-related diseases; kidney disorders; primary hyperoxaluria; primary hyperoxaluria type 1; primary hyperoxaluria type 2; primary hyperoxaluria type 3; renal disease	SC	Undisclosed	GalNAc
Olpasiran/AMG 890/ARO-LPA [82,83]	Ongoing	Liver (LPA mRNA)	Atherosclerotic cardiovascular disease (ASCVD); cardiovascular diseases; elevated serum lipoprotein(a); hepatic impairment; myocardial infarction; renal impairment	SC	Undisclosed	Targeted RNAi Molecule (TRiM^TM^) platform (GalNac)
ARO-APOC3 [32]	Ongoing	Liver (APOC3 mRNA)	Dyslipidemia; familial chylomicronemia syndrome (FCS); hypertriglyceridemia	SC	Undisclosed	Targeted RNAi Molecule (TRiM^TM^) platform
Fazirsiran/ADS-001/ARO-AAT/TAK-999 [32,82]	Ongoing	Liver (SERPINA1 mRNA)	Alpha-1 antitrypsin deficiency; liver fibrosis; PiZZ alpha-1 antitrypsin deficiency (ZZ type alpha-1 antitrypsin deficiency)	SC	Undisclosed	Targeted RNAi Molecule (TRiM^TM^) platform (GalNac)
Cemdisiran/ALN-CC5 [84,85,86]	Ongoing	Liver (C5 mRNA)	Anemia; atypical hemolytic uremic syndrome (aHUS); immunoglobulin A nephropathy (Berger disease); myasthenia gravis; paroxysmal nocturnal hemoglobinuria (PNH); renal impairment; thrombocytopenia; tuberculous meningitis	SC	Undisclosed	GalNAc
QPI-1007 [32,87]	Ongoing	Eye (CASP2 mRNA)	Glaucoma; non-arteritic anterior ischemic optic neuropathy (NAION)	IVt	Naked RNA	NA
Fitusiran */ALN-AT3SC/SAR439774 [87,88]	Ongoing	Liver (antithrombin III mRNA)	Hemophilia; hemophilia A; hemophilia B	SC	Undisclosed	GalNAc

*: These products have been approved since the data for this review were collected.

**Table 5:** Approved ASOs and structural characteristics.

INN Name	Approv.	Target Site (Target)	Disease	Posology	Deliv. Route	Backbone Mod.	2′ Mod.	5′/3′ Mod.
Casimersen (Amondys 45)	FDA 2021	Muscle fibers (DMD Exon 45)	Duchenne muscular dystrophy	30 mg/kg q.w.	IV	PMO	NA	Short PEG tail
Eteplirsen (Exondys 51)	FDA 2016	Muscle fibers (DMD Exon 51)	Duchenne muscular dystrophy	30 mg/kg q.w.	IV	PMO	NA	Short PEG tail
Golodirsen (Vyondys 53)	FDA 2019	Muscle fibers (DMD Exon 53)	Duchenne muscular dystrophy	30 mg/kg q.w.	IV	PMO	NA	Short PEG tail
Milasen [104]	FDA 2017	SNC (CLN7)	Batten’s disease (patient-customized)	42 mg quarterly after induction phase	ITh	PS	2′-O-MOE	NA
Nusinersen (Spinraza)	FDA 2016 EMA 2017	SNC (SMN2 pre-mRNA)	Spinal muscular atrophy	12 mg every four months after an induction phase	ITh	PS	2′-O-MOE	NA
Viltolarsen (Viltepso) [105]	FDA 2020	Muscle fibers (DMD Exon 53)	Duchenne muscular dystrophy	80 mg/kg q.w.	IV	PMO	NA	NA
Fomivirsen (Vitravene)	FDA 1998 EMA 1999	Eye (CMV mRNA)	Cytomegalovirus retinitis	0.165/0.330 mg/eye q.2w after induction phase	IVt	PS	NA	NA
Inotersen (Tegsedi)	FDA 2018 EMA 2018	Liver (TTR mRNA)	Hereditary transthyretin-mediated amyloidosis	285 mg q.w.	SC	PS	2′-O-MOE	NA
Mipomersen (Kynamro)	FDA 2013	Liver/intestine (apo-B-100 mRNA)	Familial hypercholesterolemia	200 mg q.w.	SC	PS	2′-O-MOE	NA
Volanesorsen (Waylivra)	EMA 2019	Liver/intestine (apoC-III mRNA)	Familial chylomicronemia	285 mg q.2w after induction phase	SC	PS	2′-O-MOE	NA

**Table 6:** Approved siRNAs and structural characteristics.

INN Name	Approv.	Target Site (Target)	Disease	Posology	Deliv. Route	Backbone Mod.	2′ MOD.	5′/3′ Mod.
Givosiran (Givlaari) [35]	FDA 2019 EMA 2020	Liver/hepatocytes (ALS1 mRNA)	Acute hepatic porphyria	2.5 mg/kg q.m.	SC	PS	2′-F, 2′-Ome	NA
Inclisiran (Leqvio) [32,36]	EMA 2020	Liver/hepatocytes (PCSK9 mRNA)	Primary hypercholesterolemia	284 mg every six months after induction phase	SC	PS	2′-F, 2′-Ome	NA
Lumasiran (Oxlumo) [37]	FDA 2020 EMA 2020	Liver/hepatocytes (HAO1 mRNA)	Primary hyperoxaluria	3 to 6 mg/kg quarterly after induction phase	SC	PS	2′-F, 2′-Ome	NA
Vutrisiran (Amvuttra) [38,39,40]	FDA 2022 EMA 2022	Liver/hepatocytes (TTR mRNA)	hATTR amyloidosis	25 mg quarterly	SC	PS	2′-F, 2′-Ome	NA
Patisiran (Onpattro)	FDA 2018 EMA 2018	Liver (TTR mRNA)	hATTR amyloidosis	0.3 mg/kg every three weeks	IV	NA	2′-Ome	NA

**Table 7:** ASOs in the late clinical phase and structure characteristics.

INN Name	Clinical Trial Status	Target Site (Target)	Disease	Deliv. Route	Backbone Mod.	2′ Mod.	5′/3′ Mod.
Tofersen */BIIB067/IONIS-SOD1Rx/ISIS 333611/BIIB067/IONIS-SOD1Rx/ISIS 333611 [52]	Ongoing	CNS (SOD1)	Amyotrophic lateral sclerosis (ALS); superoxide dismutase 1-amyotropic lateral sclerosis (SOD1-ALS)	ITh	PS	2′-O-MOE	NA
Tominersen/IONIS-HTTRx/ISIS 443139/RG6042/RO7234292 [52,53]	Ongoing	CNS (HTT)	Huntington’s disease	ITh	PS	2′-O-MOE	NA
Zilganersen/ION-1166998/ION373 [54]	Ongoing	CNS (GFAP)	Alexander disease	ITh	PS	2′-O-MOE	NA
Sepofarsen/QR-110 [55]	Ongoing	Eye (CEP290)	Blindness; congenital eye disorders; eye disorders; Leber congenital amaurosis (LCA); Leber congenital amaurosis 10 (LCA10); neurological disorders; retinal degeneration; retinal disease; retinal dystrophy; sensation disorders; vision disorders	IO	PS	2′-O-Me	NA
OT-101/AP 12009/AP-12009/ATB-301/TASO-001/TGF-β2 targeting anti-sense oligonucleotide/Trabedersen [56]	Ongoing	Tumor (TGF-B2)	Advanced malignant pleural mesothelioma; advanced solid tumors; cancer indications; colorectal cancer; diffuse intrinsic pontine glioma; glioma; melanoma; metastatic solid tumors; pancreatic cancer; pleural malignant mesothelioma; progressive myopia; SARS-CoV-2 (COVID-19) infection; solid tumors	IT	PS	NA	NA
Drisapersen/GSK2402968/Kyndrisa/PRO051 [57,58]	Stopped	Muscles (DMD exon 51)	Duchenne muscular dystrophy (DMD); muscular dystrophies	SC	PS	2′-O-MOE	NA
Ultevursen/QR-421a [59,60]	Ongoing	Eye (USH2A exon 13)	Blindness; congenital eye disorders; deafness; eye disorders; hereditary conditions; retinal disease; retinitis pigmentosa; Usher syndrome type 2; Usher syndrome type 2a; vision disorders	IO	PS	2′-O-MOE	NA
Bepirovirsen/GSK3228836/IONIS-HBVRx/ISIS 505358/ISIS-GSK3Rx/ISIS-HBVRx [61]	Ongoing	Liver (HBsAg)	Hepatic impairment; hepatitis B; liver cirrhosis; liver disorders	SC	PS	2′-O-MOE	NA
Mongersen/GED-0301 [62]	Ongoing	GI epithelial cells (SMAD7)	Crohn’s disease; ulcerative colitis	Oral	PS	NA	NA
IONIS-PKK-LRx/Donidalorsen/ISIS 721744 [63,64]	Ongoing	Liver (KLKB1)	Cardiovascular diseases; hereditary angioedema; SARS-CoV-2 (COVID-19) infection	SC	PS	2′-O-MOE	NA
Eplontersen */AKCEA-TTR-LRx/ION-682884/IONIS-TTR-LRx/ISIS-TTR(Rx) [65,66]	Ongoing	Liver (TTR mRNA)	Amyloidosis; familial amyloid polyneuropathy (hereditary transthyretin-mediated amyloid polyneuropathy); transthyretin amyloid cardiomyopathy	SC	PS	2′-O-MOE	NA
ION363/Jacifusen [67]	Ongoing	CNS (FUS)	Amyotrophic lateral sclerosis (ALS)	ITh	PS	2′-O-MOE	NA
Custirsen/OGX-011/TV-1011 [68,69]	Stopped	Tumor (sCLU)	Adenocarcinoma of the prostate; advanced non-small cell lung cancer (NSCLC); cancer indications; cardiac conduction and repolarization; castrate prostate cancer; metastatic castrate prostate cancer; metastatic non-small cell lung cancer; prostate cancer; solid tumors	IV	PS	2′-O-MOE	NA
Pelacarsen/AKCEA-APO(a)-LRx/ASO 144367/IONIS-APO(a)-LRx/ISIS 681257/TQJ230 [70]	Ongoing	Liver (LPA)	Acute coronary syndrome; cardiovascular diseases; coronary artery disease (coronary heart disease); elevated serum lipoprotein(a); genetic polymorphisms; hepatic impairment; hyperlipoproteinemia (hyperlipidemia, lipoproteinemia); inflammation; ischemic stroke; liver cirrhosis; myocardial infarction; peripheral arterial disease (PAD); renal impairment; stenosis	SC	PS	2′-O-MOE	NA
Suvodirsen/WVE-210201 [71]	Stopped	Muscles (DMD)	Duchenne muscular dystrophy	IV	PS	2′-O-Me, 2′-F	NA
Aganirsen/GS-101 [72,73]	Ongoing	Eye (IRS1)	Ischemic central retinal vein occlusion; neovascular glaucoma	Eye, topical	PS	NA	NA
Alicaforsen/ISIS-2302 [74]	Ongoing	Colon (undisclosed)	Inflammatory bowel disease; pouchitis; ulcerative colitis	Rectal	PS	NA	NA
Olezarsen */AKCEA-APOCIII-LRx/IONIS APOCIII-LRx/ISIS 678354 [75,76,77]	Ongoing	Liver (APOC3)	Atherosclerosis; atherosclerotic cardiovascular disease (ASCVD); cardiovascular diseases; coronary artery disease (coronary heart disease); elevated triglycerides; familial chylomicronemia syndrome (FCS); familial lipoprotein lipase deficiency (hyperlipoproteinemia type I); hypertriglyceridemia; ischemic stroke; pancreatitis; peripheral arterial disease (PAD)	SC	PS	2′-O-MOE	NA

*: These products have been approved since the data for this review were collected.

**Table 8:** siRNAs in the late clinical phase and structural characteristics.

INN Name	Clinical Trial Status	Target Site (Target)	Disease	Deliv. Route	Backbone Mod.	2′ Mod.	5′/3′ Mod.
Tivanisiran/SYL1001 [80]	Ongoing	Eye (TRPV1 mRNA)	Dry eye disease; ocular pain; Sjögren’s syndrome	Eye, topical	NA	NA	NA
Nedosiran */DCR-PHXC [81]	Ongoing	Hepatocytes (Liver) (LDHA mRNA)	Genetic-related diseases; kidney disorders; primary hyperoxaluria; primary hyperoxaluria type 1; primary hyperoxaluria type 2; primary hyperoxaluria type 3; renal disease	SC	PS	2′-O-Me, 2′-F	NA
Olpasiran/AMG 890/ARO-LPA [82,83]	Ongoing	Hepatocytes (Liver) (LPA mRNA)	Atherosclerotic cardiovascular disease (ASCVD); cardiovascular diseases; elevated serum lipoprotein(a); hepatic impairment; myocardial infarction; renal Impairment	SC	PS	2′-F, 2′-O-Me	NA
ARO-APOC3 [32]	Ongoing	Liver (APOC3 mRNA)	Dyslipidemia; familial chylomicronemia syndrome (FCS); hypertriglyceridemia	SC	PS	PS, 2′-OMe, 2′-F	Inverted base
Fazirsiran/ADS-001/ARO-AAT/TAK-999 [32,82]	Ongoing	Liver (SERPINA1 mRNA)	Alpha-1 antitrypsin deficiency; liver fibrosis; PiZZ alpha-1 antitrypsin deficiency (ZZ type alpha-1 antitrypsin deficiency)	SC	PS	PS, 2′-OMe, 2′-F, inverted base	Inverted base
Cemdisiran/ALN-CC5 [84,85,86]	Ongoing	Liver (C5 mRNA)	Anemia; atypical hemolytic uremic syndrome (aHUS); immunoglobulin A nephropathy (Berger disease); myasthenia gravis; paroxysmal nocturnal hemoglobinuria (PNH); renal impairment; thrombocytopenia; tuberculous meningitis	SC	PS	2′-O-Me, 2′-F, PS	NA
QPI-1007 [32,87]	Ongoing	Eye (CASP2 mRNA)	Glaucoma; non-arteritic anterior ischemic optic neuropathy (NAION)	IVt	NA	2′-O-Me,	5′ inverted deoxybasic sense
Fitusiran */ALN-AT3SC/SAR439774 [87,88]	Ongoing	Liver (AT III mRNA))	Hemophilia; hemophilia A; hemophilia B	SC	PS	2′-O-Me, 2′-F	NA

*: These products have been approved since the data for this review were collected.

**Table 9:** Delivery-related data for approved mRNA products.

INN Name	Approv.	Target Site	Indication	Posology	Deliv. Route	Deliv. System	2′/Backbone Mod.	5′/3′ Mod.	Base Mod.
Elasomeran (Spikevax) [140]	FDA 2020 EMA 2020	Undisclosed	COVID-19 vaccine	0./0.2 mg two doses 28 days apart	IM	LNP	Undisclosed	5′-cap, Poly A tail	N1-methylpseudouridine
Tozinameran (Comirnaty) [141]	FDA 2020 EMA 2020	Undisclosed	COVID-19 vaccine	0.03 mg 2 doses at least 21 days apart	IM	LNP	Undisclosed	5′-cap2, Poly A tail	N1-methylpseudouridine

**Table 10:** Delivery-related data for late clinical-phase mRNA products.

INN Name	Clinical Trial Status	Target Site	Indication	Deliv. Route	Deliv. System	2′/Backbone Mod.	5′/3′ Mod.	Base Mod.
BNT162b1 [142]	Ongoing	Undisclosed	Respiratory disease; SARS-CoV-2 (COVID-19) infection; viral diseases	IM	LNP	Undisclosed	Undisclosed	N1-Methylpseudouridine
mRNA-1273.213 [143,144,145]	Ongoing	Undisclosed	SARS-CoV-2 (COVID-19) infection	IM	LNP	Undisclosed	Undisclosed	N1-Methylpseudouridine
RQ3013 [146]	Ongoing	Undisclosed	SARS-CoV-2 (COVID-19) infection	IM	LNP	Undisclosed	Undisclosed	N1-Methylpseudouridine
LVRNA009 [147,148,149]	Ongoing	Undisclosed	SARS-CoV-2 (COVID-19) infection	IM	Undisclosed	Undisclosed	Undisclosed	LNP
Pfizer BioNtech Omicron BA.1-adapted bivalent COVID-19 vaccine	Approved; ongoing	Undisclosed	SARS-CoV-2 (COVID-19) infection	Undisclosed	Undisclosed	Undisclosed	Undisclosed	LNP
Oslo University Hospital tumor (glioblastoma) stem cells/hTERT/Survivin mRNA transfected autologous DCs [150]	Ongoing	Ex vivo cell therapy	Glioblastoma multiforme	ID	Undisclosed	Undisclosed	Undisclosed	LNP
Elasomeran/Imelasomeran/Imelasomeran/mRNA-1273.529/Spikevax Bivalent Original/Omicron BA.1	Ongoing	Undisclosed	SARS-CoV-2 (COVID-19) infection	Undisclosed	Undisclosed	Undisclosed	N1-Methylpseudouridine	LNP
DS-5670/Daiichi Sankyo COVID-19 mRNA vaccine [151]	Ongoing	Undisclosed	SARS-CoV-2 (COVID-19) infection	IM	Undisclosed	Undisclosed	Uridine-modified	LNP
ABO1020 [152]	Ongoing	Undisclosed	SARS-CoV-2 (COVID-19) infection	IM	Undisclosed	Undisclosed	Undisclosed	LNP
Rocapuldencel-T/AGS-003/AGS-003-LNG/CoImmune CMN-001 [153]	Ongoing	Ex vivo cell therapy	Advanced renal cell carcinoma; clear cell renal cell carcinoma; infiltrating bladder urothelial carcinoma; kidney cancer; metastatic kidney cancer; metastatic renal cell carcinoma; muscle-invasive bladder cancer; non-small cell lung cancer (NSCLC); renal cancer; renal cell carcinoma (RCC); urothelial carcinoma of the bladder; urothelial cell carcinoma	SC	Undis-closed	Undisclosed	Undisclosed	Electroporation; naked RNA
mRNA-1273.211 [143]	Ongoing	Undisclosed	SARS-CoV-2 (COVID-19) infection	IM	Undis-closed	Undisclosed	N1-Methylpseudouridine	LNP
Pfizer Quadrivalent Influenza modRNA Vaccine [154]	Ongoing	Undisclosed	Influenza; SARS-CoV-2 (COVID-19) Infection	IM	Undisclosed	Undisclosed	modRNA	Undisclosed
BNT161 [155,156,157]	Ongoing	Undisclosed	Influenza; SARS-CoV-2 (COVID-19) infection	IM	Undis-closed	Undisclosed	modRNA	LNP
mRNA-1010	Ongoing	Undisclosed	Cytomegalovirus disease (CMV disease); influenza; respiratory syncytial virus (RSV) infection; SARS-CoV-2 (COVID-19) infection	IM	Undis-closed	Undisclosed	Undisclosed	LNP
mRNA-1647	Ongoing	Undisclosed	Cytomegalovirus disease (CMV disease); cytomegalovirus infections; influenza; respiratory syncytial virus (RSV) infection	IM	Undis-closed	Undisclosed	Undisclosed	LNP
mRNA-1273.617.2 [143,158]	Ongoing	Undisclosed	SARS-CoV-2 (COVID-19) infection	IM	Undisclosed	Undisclosed	N1-Methylpseudouridine	LNP
Imelasomeran/elasomeran/mRNA-1273.214 [143,159]	Approved; ongoing	Undisclosed	Asthma; cystic fibrosis (CF); diabetes mellitus; HIV infection; influenza; Middle East respiratory syndrome (MERS, MERS-CoV); respiratory syncytial virus (RSV) infection; SARS-CoV-2 (COVID-19) infection	IM	Undis-closed	Undisclosed	N1-Methylpseudouridine	LNP
Walvax COVID-19 Vaccine/ARCoV/ARCoV mRNA vaccine/AWcorna [22,160]	Approved; ongoing	Undisclosed	Pneumonia; SARS-CoV-2 (COVID-19) infection; viral pneumonia	IM	NA	NA	NA	LNP
mRNA-1345 [161]	Ongoing	Undisclosed	Chronic obstructive pulmonary disease; congestive heart failure (CHF); cytomegalovirus disease (CMV disease); influenza; respiratory syncytial virus (RSV) infection; SARS-CoV-2 (COVID-19) infection	IM	Undis-closed	Undisclosed	Nucleoside-modified	LNP
PTX-COVID19-B [162]	Ongoing	Undisclosed	SARS-CoV-2 (COVID-19) infection	IM	Undis-closed	Undisclosed	Undisclosed	LNP
SW-BIC-213 mRNA vaccine [163,164,165,166]	Ongoing	Undisclosed	SARS-CoV-2 (COVID-19) infection	IM	Undisclosed	Undisclosed	Undisclosed	Lipopolyplex
CVnCoV/CureVac COVID-19 mRNA vaccine/CV07050101 [167]	Discontinued (clinical)	Undisclosed	Respiratory disease; SARS-CoV-2 (COVID-19) infection	IM	NA	NA	NA	LNP
SYS6006 [168,169]	Ongoing	Undisclosed	SARS-CoV-2 (COVID-19) infection	IM	Undisclosed	Undisclosed	Undisclosed	LNP

**Table 11:** Approved aptamers and structural characteristics.

INN Name	Approv.	Target Site (Target)	Disease	Posology	Delivery Route	Backbone Mod.	2′ Mod.	5′/3′ Mod.
Defibrotide (Defitelio) [192]	FDA 2016 EMA 2013	Liver endothelium/blood (adenosine A1/A2 receptor)	Sinusoidal obstruction syndrome	25 mg/kg daily, up to 6 week	IV	Undisclosed	Undisclosed	Undisclosed
Pegaptanib (Macugen)	FDA 2004	Eye (heparin-binding domain of VEGF-165)	Age-related macular degeneration	0.3 mg every six months	IVt	NA	2′-O-Me, 2′-F,	3′-inverted nucleotide; PEG conjugate

**Table 12:** Aptamers in the late clinical phase and structural characteristics.

INN Name	Clinical Trial Status	Target Site	Disease	Deliv. Route	Backbone Mod.	2′ Mod.	5′/3′ Mod.
Pegnivacogin/REG1 (RB006) [193,194]	Ongoing	Blood (factor IXA)	Angina (angina pectoris); coronary artery disease (coronary heart disease)	IV	NA	2′-F, 2′-O-Me	3′ inverted deoxythymidine; PEG conjugate
Fovista^®^/E10030, Pegpleranib [195]	Ongoing	Eye (PDGF)	Choroidal neovascularization; dry age-related macular degeneration (atrophic macular degeneration); neovascular age-related macular degeneration (wet age-related macular degeneration)	IO	PEG loops	2′-F, 2′-O-Me	3′ inverted deoxythymidine; PEG conjugate
Zimura/Avacincaptad pegol [116,196]	Ongoing	Eye (C5 inhibitor)	Age-related macular degeneration (AMD); cancer indications; choroidal neovascularization; dry age-related macular degeneration (atrophic macular degeneration); hereditary ATTR amyloidosis; idiopathic polypoidal choroidal vasculopathy; macular degeneration; neovascular age-related macular degeneration (wet age-related macular degeneration); Stargardt macular dystrophy (Stargardt disease, fundus flavimaculatus)	IVt	NA	2′-O-Me	3′ inverted deoxythymidine; PEG conjugate

**Table 13:** Approved aptamers and drug delivery-relevant characteristics.

INN Name	Approval(s)	Target Site (Target)	Disease	Posology	Deliv. Route	Deliv. System	Conjugation/Termination
Defibrotide (Defitelio)	FDA 2016 EMA 2013	Liver endothelium/blood (Adenosine A1/A2 receptor)	Sinusoidal obstruction syndrome	25 mg/kg daily, up to 6 week	IV	Naked in citrate buffer	NA
Pegaptanib (Macugen)	FDA 2004	Eye (Heparin-binding domain of VEGF-165)	Age-related macular degeneration	0.3 mg every six months	IVt	Naked in NaCl phosphate buffer	NA

**Table 14:** Aptamers in the late clinical phase and drug delivery relevant characteristics.

INN Name	Clinical Trial Status	Target Site (Target)	Disease	Deliv. Route	Deliv. System	Conjugation/Termination
Pegnivacogin/REG1 (RB006) [193,194]	Ongoing	Blood (Factor IXA)	Angina (angina pectoris); coronary artery disease (coronary heart disease)	IV	Undisclosed	NA
Fovista^®^/E10030, Pegpleranib [195]	Ongoing	Eye (PDGF)	Choroidal neovascularization; dry age-related macular degeneration (atrophic macular degeneration); neovascular age-related macular degeneration (wet age-related macular degeneration)	IO	Undisclosed	NA
Zimura/Avacincaptad pegol [116,196]	Ongoing	Eye (C5 inhibitor)	Age-related macular degeneration (AMD); cancer indications; choroidal neovascularization; dry age-related macular degeneration (atrophic macular degeneration); hereditary ATTR amyloidosis; idiopathic polypoidal choroidal vasculopathy; macular degeneration; neovascular age-related macular degeneration (wet age-related macular degeneration); Stargardt macular dystrophy (Stargardt disease, fundus flavimaculatus)	IVt	Undisclosed	NA

## Abbreviations

The following abbreviations are used in this manuscript:

MDPI	Multidisciplinary Digital Publishing Institute
DOAJ	Directory of open access journals
ASO	Antisense oligonucleotide
ASGPR	Asialoglycoprotein receptor
circRNA	Circular RNA
CRISPR	Clustered regularly interspaced short palindromic repeats
DDS	Drug delivery system
DMD	Duchenne muscular dystrophy
DNA	Deoxyribonucleic acid
EMA	European Medicines Agency
EV	Extracellular vesicle
FDA	Food and Drug Administration
GalNac	N-acetylgalactosamine
LNP	Lipid nanoparticle
RNA	Ribonucleic acid
mRNA	Messenger RNA
miRNA	Micro-RNA
ORF	Open Reading Frame
PMO	Phosphorodiamidate morpholino-oligomer
PS	Phosphorothioate
RISC	RNA-induced silencing complex
saRNA	Self-amplifying RNA
siRNA	Small interfering RNA
sgRNA	Single guide RNA
UTR	Untranslated region
2′-F	2′-Fluororibonucleotides
2′-OMe	2′-O-methyl-ribonucleotides
2′-O-MOE	2‘-O-(2-Methoxyethyl)-ribonucleotides

## Appendix A

**Table A1:** Late clinical-phase nucleic acid products outside of the main four classes presented above drug delivery relevant characteristics.

Modality	Drug Names	Clinical Trial Status	Deliv. Route	Nucleic Acid Modifications	Target Site (Target)	Deliv. System	Disease Indication
Oligonucleotide	Lefitolimod/GS-1703/MGN1703 [207,208]	Ongoing	SC	Fully natural	Undisclosed (TLR9 agonist)	Undisclosed	Acquired immunodeficiency syndrome (AIDS); advanced solid tumors; extensive-stage (ES) small cell lung cancer (SCLC); HIV infection; melanoma; metastatic colorectal cancer; metastatic solid tumors; neuroendocrine tumors of pancreatic origin; peritoneal cancer; small cell lung cancer (SCLC)
	Cobitolimod [209]	Ongoing	Rectal	PS	Undisclosed (TLR9 agonist)	Undisclosed	Severe ulcerative colitis; ulcerative colitis
	Imetelstat/GRN163L/Imetelstat sodium [210]	Ongoing	IV	Thiophosphoramidate (NPS), lipid conjugate (for permeability)	Undisclosed (telomerase inhibitor)	Undisclosed	Acute myeloid leukemia; adenocarcinoma of the breast; adult giant cell glioblastoma; advanced non-small cell lung cancer (NSCLC); anaplastic astrocytoma; anaplastic ependymoma; anaplastic oligodendroglioma; astrocytoma; bone marrow metastatic disease; brain cancer; brain tumor; brainstem glioma; brainstem tumor; breast adenocarcinoma; breast neoplasm; central nervous system tumors; cytopenia; diffuse intrinsic pontine glioma; ependymoma; essential thrombocythemia; Ewing’s sarcoma; glioblastoma multiforme; gliosarcoma; hepatoblastoma; HER2-positive breast cancer; high-grade glioma; liver cancer; lymphoma; lymphoproliferative disease; malignant solid tumor; metastatic breast cancer; metastatic non-small cell lung cancer; myelodysplastic syndrome; myelofibrosis; myeloid malignancies; myeloma/multiple myeloma/Kahler’s disease/myelomatosis; myeloproliferative neoplasm and myelodysplastic syndrome; neuroblastoma; non-secretory myeloma; non-small cell lung cancer (NSCLC); oligodendroglioma; osteosarcoma; peripheral primitive neuroectodermal tumor; polycythemia vera; post-essential thrombocythemia (Post-ET) myelofibrosis; post-polycythemia vera myelofibrosis; post-transplant lymphoproliferative disorders (PTLD); primary myelofibrosis; rhabdomyosarcoma; cecondary myelofibrosis; secretory multiple myeloma; small intestine lymphoma; solid tumors
gRNA	Exa-cel/CTX001/Exagamglogene autotemcel [211]	Ongoing	IV	Undisclosed	Hematopietic stem cells (BCL11A)	Ex vivo—electroporation	Anemia; beta thalassemia; genetic-related diseases; hematological disease; hemoglobinopathy; sickle cell anemia; sickle cell diseases; thalassemia; transfusion-dependent β-thalassemia
	LBP-EC01/crPhage^TM^ [212,213]	Ongoing	Intraurethral/IV	Undisclosed	Vesical *E. coli*	In vivo—bacteriophage	Urinary tract infection
saRNA	ARCT-154	Ongoing	IM	Undisclosed	Undisclosed	Undisclosed	SARS-CoV-2 (COVID-19) infection
	GRANITE-001/GRT-C901 (Prime)/GRT-R902 (Boost) [214,215]	Ongoing	IM	Undisclosed	Undisclosed (neoantigen)	ChAd68 LNP; Replicon RNA (samRNA) (adenovirus)	Colorectal cancer; colorectal neoplasms; gastroesophageal adenocarcinoma; metastatic cancer; metastatic colorectal cancer; metastatic gastroesophageal adenocarcinoma; metastatic microsatellite-stable colorectal cancer; metastatic non-small cell lung cancer; metastatic urothelial carcinoma; microsatellite-stable colorectal cancer; non-small cell lung cancer (NSCLC); solid tumors; urothelial cancer
shRNA	Vigil/FANG Immunotherapy/Gemogenovatucel-T/Vigil^TM^ Vaccine [216]	Clinically active	ID	Undisclosed	Undisclosed (neoantigen)	Undisclosed	Advanced breast cancer; advanced cervical cancer; advanced endometrial cancer; advanced fallopian tube cancer; advanced gynecologic cancer; advanced melanoma; advanced non-small cell lung cancer (NSCLC); advanced ovarian cancer; advanced peritoneal cancer; advanced solid tumors; advanced uterine cancer; bone tissue neoplasm; breast cancer; cervical cancer; clear cell epithelial ovarian cancer; colorectal carcinoma; connective tissue neoplasm; endometrial cancer; endometrioid epithelial ovarian cancer; endometrioid ovarian cancer; Ewing’s sarcoma; Ewing’s tumor; fallopian tube cancer; high-grade serous ovarian cancer; large bowel (colon) cancer; liver cancer; lung cancer; melanoma; metastatic breast cancer; metastatic cervical cancer; metastatic endometrial cancer; metastatic Ewing sarcoma; metastatic Ewing’s tumor; metastatic fallopian tube cancer; metastatic melanoma; metastatic non-small cell lung cancer; metastatic ovarian cancer; metastatic peritoneal cancer; metastatic solid tumor; metastatic uterine cancer; neoplasms; non-small cell lung cancer (NSCLC); ovarian cancer; ovarian clear cell carcinoma; ovarian neoplasm; papillary ovarian cancer; peritoneal cancer; peritoneal neoplasms; primary peritoneal carcinoma; rare tumors; sarcoma; serous epithelial ovarian cancer; serous ovarian cancer; soft tissue neoplasms; solid tumors; uterine cancer; uterine papillary serous carcinoma
